# TRIM25 facilitates mitochondrial dysfunction and extracellular matrix degradation by enhancing USP7‐driven ENO1 deubiquitination during intervertebral disc degeneration

**DOI:** 10.1002/ctm2.70785

**Published:** 2026-08-31

**Authors:** Xiaoming Liu, Wenyu Zhang, Hang Feng, Linying Lai, Chen Cao, Guang Yang, Yanzheng Gao, Bijun Wang, Hui Wang, Bin Yu, Zhenghong Yu

**Affiliations:** ^1^ Department of Orthopedics Tongren Hospital Shanghai Jiao Tong University School of Medicine Shanghai China; ^2^ Center for Spinal Minimally Invasive Research Shanghai Jiao Tong University Shanghai China; ^3^ Department of Spine Surgery Shanghai East Hospital School of Medicine Tongji University Shanghai China; ^4^ Department of Orthopaedics Navy 905th Hospital Shanghai China; ^5^ Department of Surgery of Spine and Spinal Cord Henan Provincial People's Hospital Zhengzhou China; ^6^ Department of Gastroenterology and Hepatology Institute of Digestive Disease Tongji Hospital School of Medicine Tongji University Shanghai China

**Keywords:** TRIM25, ENO1, USP7, intervertebral disc degeneration, ubiquitin‐specific protease 7

## Abstract

**Background:**

The pathology of intervertebral disc degeneration (IDD) is characterized by metabolic dysregulation within nucleus pulposus (NP) cells. TRIM25 has been implicated in diverse tumors and pathological processes, yet its precise role in mediating mitochondrial function and metabolic alterations during IDD progression remains unclear.

**Methods:**

Transcriptome sequencing was performed to analyze gene expression changes during IDD progression. Molecular biology techniques including co‐immunoprecipitation and ubiquitination assays were used to investigate the interaction between TRIM25 and the glycolytic enzyme ENO1 and its regulatory mechanism. Cellular and in vivo animal models were employed to validate the effects of the TRIM25‐USP7‐ENO1 axis on glycolysis, mitochondrial function, ATP levels, and extracellular matrix degradation.

**Results:**

Transcriptome sequencing revealed that glycolysis‐related pathways and TRIM25 were significantly upregulated during IDD progression. Mechanistically, TRIM25 interacted with ENO1. Contrary to its typical E3 ligase function, TRIM25 overexpression stabilized ENO1 by reducing its K48‐linked polyubiquitination. Furthermore, TRIM25 enhanced the interaction between USP7 and ENO1, leading to USP7‐mediated deubiquitination and stabilization of ENO1. Disruption of the TRIM25‐USP7‐ENO1 axis suppressed glycolysis, improved mitochondrial function, elevated ATP levels, and inhibited extracellular matrix degradation. In vivo, modulation of this axis correspondingly accelerated or ameliorated IDD progression.

**Conclusion:**

We identified a novel TRIM25‐USP7‐ENO1 axis, through which TRIM25 stabilizes ENO1 by promoting USP7‐ENO1 interaction and subsequent USP7‐dependent deubiquitination. This non‐canonical function expands the known role of TRIM25 and highlights a promising therapeutic target for restoring mitochondrial dysfunction and metabolic homeostasis in IDD.

**Key points:**

- TRIM25 recruits the deubiquitinating enzyme USP7 to collaboratively enhance the deubiquitination and stability of the key glycolytic enzyme ENO1, establishing a new regulatory pathway linking inflammation and metabolism.
- This regulatory axis exacerbates glycolysis, impairs mitochondrial function, disrupts cellular energy homeostasis, and ultimately leads to extracellular matrix degradation, systematically explaining a new pathological mechanism of IDD.
- Both cellular and animal models confirm that intervention in the TRIM25/USP7/ENO1 axis effectively reverses metabolic imbalance and degenerative progression, offering a promising new therapeutic strategy for IDD.
- This study extends TRIM25's role from immune regulation to metabolic processes and is the first to report USP7's involvement in glycolytic enzyme regulation, providing a new paradigm for studying “non‐canonical” functions of E3 ligases and deubiquitinating enzymes.

## Graphical abstract

1. Under conditions of low TRIM25 expression, the ubiquitination fate of ENO1 shifts to K48‐linked ubiquitination and degradation.
2. TRIM25 enhances the deubiquitination of ENO1 by recruiting USP7, effectively inhibiting its proteasomal degradation and significantly increasing ENO1 stabilization.
3. ENO1 triggers metabolic reprogramming, shifting energy metabolism from oxidative phosphorylation to glycolysis, ultimately leading to mitochondrial dysfunction and ECM degradation.

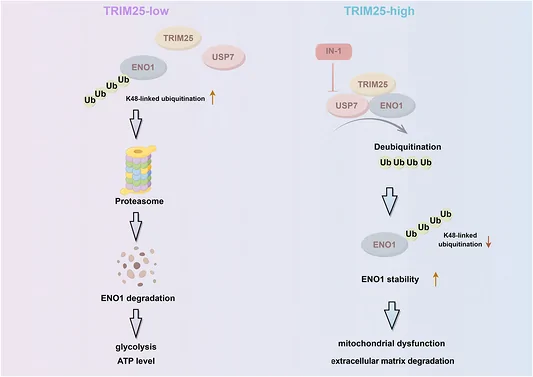

## 1 INTRODUCTION

Intervertebral disc degeneration (IDD), the leading cause of chronic low back pain, affecting hundreds of millions of people worldwide and resulting in productivity losses. Epidemiological surveys show that over 80% people will experience symptomatic low back pain, with IDD serving as the primary pathological basis.^1^, ^2^ The pathological features of IDD include apoptosis of nucleus pulposus (NP) cells, an imbalance between degradation and synthesis of the extracellular matrix (ECM) (such as collagen II (COL2) and aggrecan (ACAN)), ultimately leading to dehydration, loss of height and mechanical function in the intervertebral disc (IVD).^3^, ^4^ Traditionally, IDD has been viewed as an age‐related, passive “wear and tear” process. Recently, emerging studies indicated IDD is an active, cell‐mediated pathological process, in which metabolic disturbances of NP cells play a central role.^5^ Moreover, NP is the largest avascular tissue in the human body and relies heavily on anaerobic glycolysis to produce adenosine triphosphate (ATP).^6^, ^7^, ^8^ Therefore, disrupting glycolytic and metabolic homeostasis could become the ‘trigger’ for IDD.

Tripartite motif‐containing protein 25 (TRIM25), a key member of the TRIM family, including a RING domain mediating protein interaction, a B‐box domain, and a coiled‐coil domain.^9^, ^10^ As an E3 ubiquitin ligase, TRIM25 mainly targets substrate proteins for proteasomal degradation by catalysing K48‐linked polyubiquitination, or regulates protein activity and localization via K63‐linked ubiquitination.^11^, ^12^, ^13^ TRIM25 is widely known for innate immune responses, such as catalysing the ubiquitination of the viral RNA sensor RIG‐I to initiate the type I interferon antiviral response.^14^, ^15^, ^16^, ^17^ Recently, mounting evidence suggests that the E3 ligase family can function as a ‘scaffold’ or ‘platform’ protein, thereby regulating intricate intracellular signalling.^18^, ^19^, ^20^ In addition, TRIM25 has been reported to act an oncogene, with its function highly dependent on interacting partners.^21^, ^22^ In recent years, the role of TRIM25 in the pathological process of IDD has begun to attract interest. Studies have shown that TRIM25 can mediate stress‐induced apoptosis of nucleus pulposus (NP) cells and participates in metabolic dysregulation by regulating PARG degradation.^23^ Furthermore, TRIM25 has also been reported to be involved in the ubiquitination modification of specific proteins in senescent NP cells.^24^ These works suggest that TRIM25 may play multifaceted roles in IDD. However, its precise regulatory mechanisms and functions in the metabolic reprogramming of NP cells, particularly at the level of post‐translational modification of key glycolytic enzymes, have not yet been elucidated.

Glycolysis, one of the most ancient and conserved metabolic pathways, mainly decomposing one molecule of glucose into two molecules of pyruvate in the cytoplasm.^25^, ^26^, ^27^ In the hypoxic environment, mitochondrial oxidative phosphorylation is limited, making efficient glycolysis a ‘lifeline’ for maintaining cell survival, ECM synthesis, and intracellular homeostasis of NP cells.^6^, ^28^ Recent findings reveal that aberrant upregulation of HIF‐1α in IDD promotes glycolysis and impairs mitochondrial function, thereby increasing the risk of age‐related IDD.^29^, ^30^ Consequently, the precise role of glycolysis in IDD progression and the key enzyme in the glycolytic pathway directly determine the fate of NP cells remain to be elucidated.

α‐Enolase, encoded by the ENO1 gene, responsible for catalysing the reversible conversion of 2‐phosphoglycerate (2‐PG) to phosphoenolpyruvate (PEP) in the glycolytic pathway.^31^ ENO1 plays complex roles in cell proliferation, differentiation, survival and stress responses.^32^, ^33^, ^34^ Post‐translational modification is the main way to regulate protein function, the dynamic and reversible process of ubiquitination and deubiquitination serves as a ‘rapid switch’ regulating the turnover and stability of the protein. Notably, dysfunction of ENO1 is closely associated with various musculoskeletal diseases.^35^, ^36^ Studies have shown that in osteoarthritis, ENO1 exacerbates inflammatory responses by promoting glycolysis in synovial cells.^37^ However, these investigations have primarily focused on changes in ENO1 expression levels and its functional characterization, while little is known about the upstream molecular mechanisms underlying its aberrant activation in the pathological context of IDD, particularly the regulatory network of post‐translational modifications. Therefore, elucidating the precise regulatory mechanisms of ENO1 in NP cells holds significant value for understanding the pathological processes of metabolic disorders in IDD and developing targeted intervention strategies.

Here, we found that TRIM25 stabilizes ENO1 in degenerated NP cells, thereby affecting glycolytic flux and NP cell homeostasis. Surprisingly, our research revealed that TRIM25 mediates the stabilization of ENO1 via the ubiquitin‐specific protease 7 (USP7). Dysregulation of this axis might lead to mitochondrial dysfunction and energy metabolism disorder in IDD. This study elucidates a novel molecular mechanism of IDD and suggests that targeting TRIM25/USP7/ENO1 might become a potential therapeutic strategy to delay or even reverse the progression of IDD.

## 2 MATERIALS AND METHODS

### 2.1 Isolation, cultivation and treatment of cells

Primary NP cells were isolated from Sprague‐Dawley rats. IVD tissues were carefully dissected to isolate the NP portion. NP tissue blocks were subjected to digestion in a 0.1% Type II collagenase solution at 37°C for a duration of 4 h. After digestion was terminated, the cell suspension was collected and cultured. NP cells were treated with 10 ng/mL interleukin‐1β (IL‐1β) for 72 h to construct an in vitro degeneration model. All subsequent experiments involving IL‐1β treatment (such as WB, qPCR and metabolic assays) were conducted with a consistent treatment duration of 72 h.

### 2.2 Plasmids and Reagents

shRNA transfection targeting TRIM25 (5′‐GCTTTCGAGACGATGATTATT‐3′),^23^ ENO1(Santa Cruz Biotechnology),^38^ or USP7 genes (NM_001024790.1, Creative Biogene), the lysosomal inhibitor chloroquine (CQ, 25 µM, Cat# HY‐17589A, MCE), CHX (50 µg/mL, Sigma‐Aldrich), and MG132 (20 µM, MedChemExpress). To minimize potential off‐target effects, shRNA sequences targeting the ENO1 and USP7 mRNA, respectively, were either directly adopted or derived from previously validated constructs reported in high‐quality publications, as cited. Knockdown efficiency for each target gene was consistently verified by quantitative real‐time PCR (qPCR) and Western blot (Wb), in parallel with functional experiments. Lipofectamine 2000 served as the transfection reagent for transfecting 100 pmol of siRNA or shRNA, in line with the manufacturer's directions.

### 2.3 Wb

Cellular proteins were extracted with a strong RIPA lysis buffer containing PMSF, and their concentrations were determined using a BCA kit. The samples were then standardized, boiled, run on a 10% SDS‐PAGE gel, and transferred onto a PVDF membrane. The membrane was treated with 5% skim milk to block it, exposed to primary antibodies (antibody details in Table S1) overnight, and then incubated with HRP‐conjugated secondary antibodies for one hour. Detection was performed using an ECL chemiluminescence kit. Ultimately, ImageJ was used to quantify the grayscale values of the target bands.

### 2.4 RNA Extraction and qPCR

Cellular RNA was extracted with TRIzol, and cDNA was synthesized using the PrimeScript RT Kit. Gene expression was then measured via real‐time qPCR with GAPDH as the reference gene, using the 2^(–ΔΔCt)^ method. Primer sequences are listed in Table S2.

### 2.5 Immunofluorescence (IF)

IF staining was performed on climbing slices or sections by fixing the samples with 4% paraformaldehyde for 15 min, followed by washing with PBS. Blocking was done with 5% BSA for 30 min. Diluted primary antibodies (details in Table S1) were applied and incubated overnight at 4°C. After washing, Alexa Fluor® 488 or 594 secondary antibodies were added and incubated for 1 h in the dark. Nuclei were counterstained with DAPI for 5 min, washed, and mounted. Observations were made using a Zeiss LSM 900 confocal microscope.

### 2.6 Co‐immunoprecipitation (Co‐IP)

Cell samples were broken down in cold IP lysis buffer (Cat# P0013, Beyotime) at 4°C for 30 min, followed by centrifugation at 12 000 rpm for 15 min. The supernatant was clarified using Protein A/G Agarose beads (Bio‐Rad) at 4°C for 2 h. A part was kept as an Input control, and the remainder was incubated overnight at 4°C with the primary antibody targeting the desired protein (see Table S1 for details). On the next day, the antigen‐antibody complex was bound by adding Protein A/G Agarose beads for 4 h at 4°C. The complex underwent four washes with cold IP lysis buffer, was then combined with 1× SDS loading buffer, boiled for 10 min, centrifuged, and the supernatant was utilized for Wb detection.

### 2.7 IDD Animal Model

The study involved 3‐month‐old male SD rats, which were randomly distributed into 8 groups: Control group, IDD group (needle puncture injury), IDD + OE‐Vector, IDD + shVector, IDD+shENO1 group, IDD+OE‐TRIM25 group, IDD+OE‐TRIM25+shUSP7 group, and IDD+shUSP7 group, with 6 rats per group. The Co6/7 coccygeal disc was punctured with a 21‐gauge needle to a depth of 5 mm for 30 s to develop an IDD model. Lentiviruses for overexpression (OE‐TRIM25, OE‐Vector) and knockdown (shENO1, shUSP7, shCtrl) were constructed and packaged (GeneChem). Viral titres were 1 × 10^8^ TU/mL. All intradiscal injections were performed immediately after disc puncture. After the puncture procedure, 2 µL of the corresponding intervention reagent was immediately administered as a local injection into the same IVD space. A total volume of 2 µL containing the specified virus (or combination of viruses for co‐injection groups) was slowly injected into the centre of the NP using a 33‐gauge micro‐syringe (Hamilton). Samples were collected at 4th and 8th weeks after intervention for imaging and histological evaluation. To confirm the successful transduction and the intended effects of the lentiviral vectors in NP tissue, the expression levels of target genes (TRIM25 and USP7) were evaluated. The efficacy of overexpression and knockdown was confirmed by tissue IF staining. This experimental protocol was approved by the Tongji University Animal Experiment Ethics Committee (Approval No.: TJBB05022101).

### 2.8 Imaging and Histological Evaluation

X‐rays and a 3.0T MRI system were used to image rat tail vertebrae at 4 and 8 weeks after surgery. Intervertebral space changes were assessed by calculating the Disc Height Index (DHI%) with the formula: (D + E + F) × 100% / (A + B + C + D + E + F), where the upper vertebral body's edge heights are labelled as A, B, and C, and the intervertebral space heights are labelled as D, E and F. An index calculated from the NP area and signal intensity was used to evaluate IVD rehydration with T2‐weighted MRI. For histological analysis, the vertebrae were preserved in 4% paraformaldehyde for 48 h and then decalcified using 10% EDTA over a period of 8 weeks. After rinsing with running water, dehydration with gradient ethanol, clearing, and paraffin embedding, 4 µm thick consecutive sections were prepared. Haematoxylin‐eosin (HE) and Safranin O‐Fast Green (SO) staining were performed separately to observe IVD morphological structure and proteoglycan distribution. The degree of IDD was independently assessed by two observers blinded to the grouping information according to the Masuda scoring criteria.

### 2.9 Measurement of Mitochondrial Oxygen Consumption Rate (OCR) and Glycolytic Proton Efflux Rate (PER)

To ensure data comparability, the raw data for PER and OCR were normalized to the cell number per well. Specifically, the optimal cell seeding density was determined in a preliminary experiment prior to the formal assay. After completing the Seahorse assay, the CCK‐8 reagent was introduced to each well and left to incubate at 37°C for 2 h. A microplate reader was used to measure absorbance at 450 nm, and raw PER and OCR values were normalized to the CCK‐8 absorbance for per‐cell metabolic rates. The PER and Seahorse XF96 Mitochondrial Stress Test analysis kit measured glycolysis and mitochondrial respiration in NP cells. Cells were placed in XF96 microplates at 15 000 per well, left to incubate overnight at 37°C, and subsequently moved to Seahorse detection medium in a CO_2_‐free incubator for 60 min. Basal OCR was measured, followed by the addition of 3 µM FCCP, 2 µM oligomycin and 1 µM rotenone/antimycin A. In the glycolytic rate assessment, 0.5 µM rotenone/antimycin A and 50 mM 2‐deoxyglucose were added in order after the basal state measurement for detection. Data for OCR and PER were standardized to the number of cells and processed with WAVE software.

### 2.10 ATP Content Assay

Intracellular ATP content was measured using an Abcam ATP detection kit. Cells were lysed, centrifuged, and the supernatant was combined with ATP working solution. Luminescence was measured with a SynergyH1 microplate reader. Protein levels were measured using the BCA method, and ATP concentrations were expressed in nmol/mg protein to facilitate group comparisons.

### 2.11 Mitochondrial Tracking Staining Analysis

To explore mitochondrial morphology, NP cells were placed on cover slips coated with poly‐L‐lysine in 12‐well plates. After treatment, they were stained with 250 nM MitoTracker Red CMXRos and then examined with a confocal microscope. For quantitative analysis of mitochondrial morphology, images from at least 5 random fields per group (with >30 cells analysed in total) were acquired in a blinded manner. Scale bars are indicated on all representative images. The acquired images were analysed using the Mitochondrial Network Analysis toolset within ImageJ software to measure parameters such as the average branch length of branches per mitochondrion, which reflect the degree of mitochondrial network fragmentation.

### 2.12 RNA sequencing

RNA was isolated from rat NP cells exposed to IL‐1β or not for a duration of 72 h using TRIzol. Quality was checked with an Agilent Bioanalyzer. The preparation of libraries was done with the NEBNext Ultra RNA Library Prep Kit, followed by sequencing on an Illumina NovaSeq. Fastp processed the reads, which were then aligned to the rat genome using HISAT2. Gene expression levels were measured in FPKM using StringTie, while DESeq2 was used for differential expression analysis. Significant genes had |log2FoldChange| > 1 and adjusted *p*‐value < 0.05. KEGG and GO enrichment analyses were done with clusterProfiler.

### 2.13 Screening for deubiquitinating enzymes (DUBs)

A total of 51 DUB siRNAs were used in this screening. Forty‐eight hours post‐transfection, cell lysates were then subjected to Wb. DUBs whose knockdown led to a marked decrease in ENO1 protein levels were selected as candidate regulators. The interaction between candidate DUBs (e.g., USP7) and ENO1 or TRIM25 was further validated by reciprocal co‐immunoprecipitation assays as described in the above‐mentioned section.

### 2.14 Statistical assessment

The software GraphPad Prism 9.5.0 was used for conducting statistical analyses and plotting charts. Data are presented as mean ± standard deviation (Mean ± SD) from at least three independent experiments, with sample sizes detailed in figure legends. Normality and variance homogeneity were checked using the Shapiro–Wilk and Brown–Forsythe tests, respectively. For normally distributed and homogeneous data, an unpaired t‐test compared two groups, while one‐way ANOVA compared multiple groups, followed by the Tukey test for post‐hoc analysis if needed. Statistical significance was set at *p* <  .05, with significance levels indicated as *p < 0.05, *p* <  .01, *p <  .001, and ‘ns’ for no significant difference.

## 3 RESULTS

### 3.1 TRIM25 is upregulated in IDD and promotes NP cell degeneration by disrupting cellular energy homeostasis

**FIGURE 1:**
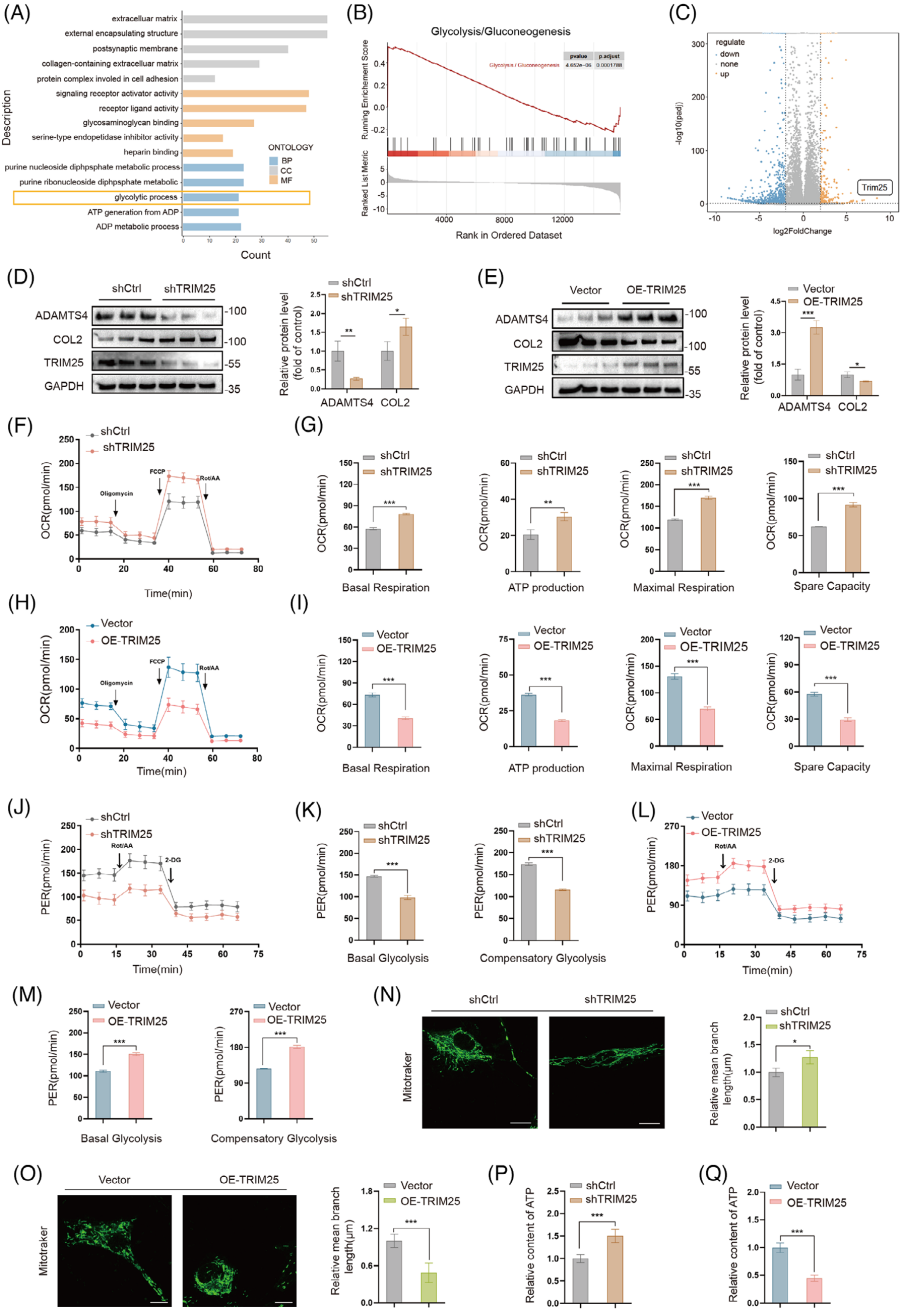
TRIM25 is upregulated in IDD and promotes NP cell degeneration by disrupting cellular energy homeostasis. (A–C) NP cells were divided into control and IL‐1β (10 ng/mL) groups, followed by transcriptomic sequencing, along with GO enrichment analysis, GSEA enrichment analysis and differential gene volcano plot analysis. (D and E) Western blot assay to detect the protein levels of TRIM25, ADAMTS4 and COL2 of NP cells transfected as indicated (*n* = 3). (F and G) OCR profiles and corresponding mitochondrial respiration parameters in shCtrl and shTRIM25 NP cells (*n* = 3). (H and I) OCR of Vector and OE‐TRIM25 NP cells, and the analysis of mitochondrial respiration (*n* = 3). (J and K) PER of shCtrl and shTRIM25 NP cells, and the analysis of basal and compensatory glycolysis (*n* = 3). (L and M) PER of Vector and OE‐TRIM25 NP cells, and the analysis of basal and compensatory glycolysis (*n* = 3). (N) Representative Mitotracker staining images depicting mitochondrial networks in shCtrl and shTRIM25 NP cells, with quantitative analysis of mean branch length (*n* = 3). Scale bars: 20 µm. (O) Representative images of Mitotracker staining to observe the mitochondrial network of Vector and OE‐TRIM25 NP cells, and the statistical analysis of mean branch length (*n* = 3). Scale bars: 20 µm. (P) ATP content quantification in shCtrl and shTRIM25 NP cells (*n* = 3). (Q) ATP content quantification in Vector and OE‐TRIM25 NP cells (*n* = 3). All data are expressed as the mean ± SD. One‐way ANOVA and Tukey's multiple comparisons test were used for statistical analysis. *p <  .05, **p <  .01, ***p <  .001. ADAMTS4, a disintegrin and metalloproteinase with thrombospondin motifs 4; ANOVA, analysis of variance; ATP, adenosine triphosphate; COL2, collagen type II; GO, Gene Ontology; GSEA, Gene Set Enrichment Analysis; IDD, intervertebral disc degeneration; IL‐1β, interleukin‐1 beta; NP, nucleus pulposus; OCR, oxygen consumption rate; PER, proton efflux rate; SD, standard deviation; TRIM25, tripartite motif containing 25.

To investigate the mechanisms underlying IDD progression, we exposed rat NP cells to IL‐1β (10 ng/mL, 72h) to recapitulate the degenerative conditions in vivo. Transcriptome sequencing revealed that the ‘Glycolysis/Gluconeogenesis’ was significantly enriched in degenerated NP cells through GO pathway (Figure 1A) and GSEA (Figure 1B) enrichment analyses. Meanwhile, qPCR analyses confirmed that the expression of glycolysis‐related genes in NP cells also increased following IL‐1β intervention (Figure S1A). At the same time, the volcano map showed that TRIM25 was a significantly different gene between the control group and the model group (Figure 1C). To validate the inflammatory stimulation model, NP cells were treated with IL‐1β (10 ng/mL, 72h) (Figure S1B,C). QPCR and Wb analyses confirmed that IL‐1β stimulation significantly upregulated the mRNA and protein levels of TRIM25, along with degeneration markers (ADAMTS4) and markedly decreasing the synthetic marker COL2, collectively indicating successful establishment of an IDD‐mimicking microenvironment.

We sought to determine the correlation between TRIM25 and NP cell degeneration, as shown in Figure 1D, knocking down TRIM25 significantly alleviated the degenerative phenotype, manifested by upregulated expression of the major ECM component COL2 and downregulated expression of the matrix‐degrading enzyme ADAMTS4. Conversely, overexpression of TRIM25 exacerbated degeneration, leading to decreased COL2 levels and increased ADAMTS4 levels (Figure 1E).

Next, we delved into the mechanism by which TRIM25 regulates NP cell glycolysis. As shown in Figure 1F,G, knocking down TRIM25 significantly enhanced the basal respiration, ATP production, maximal respiration, and spare respiratory capacity of NP cells. In contrast, overexpression of TRIM25 led to a significant decrease in these OCR parameters (Figure 1H,I), indicating that TRIM25 impairs mitochondrial oxidative phosphorylation. Moreover, TRIM25 knockdown in NP cells upregulated the expression of mitochondrial respiratory chain complex subunits and fusion markers (OPA1), while downregulating the fission protein Drp1, indicating TRIM25 enhanced mitochondrial oxidative phosphorylation (Figure S1D). Simultaneously, we assessed glycolytic flux by measuring the PER. As shown in Figure 1J,K, knockdown of TRIM25 impaired both basal and compensatory glycolytic capacity in the NP cells. Overexpression of TRIM25 promoted the glycolytic level (Figure 1L,M). These data collectively indicate that TRIM25 shifts the energy metabolism of NP cells from mitochondrial oxidative phosphorylation towards glycolysis.

Given that mitochondrial function is closely related to its morphology, we further observed morphological changes using fluorescence microscopy imaging and performed quantitative analysis. The results showed that knocking down TRIM25 promoted mitochondrial network fusion, manifested by a significant increase in the relative average branch length of the skeleton, with mitochondria presenting a healthy, elongated tubular network (Figure 1N). Consistently, the total intracellular ATP content also increased (Figure 1P). Conversely, overexpression of TRIM25 induced obvious mitochondrial fragmentation (Figure 1O), and accompanied by a significant decrease in intracellular ATP content (Figure 1Q). In summary, the results confirm that TRIM25 disrupts mitochondrial integrity, inhibits mitochondrial respiratory function, and promotes glycolysis, thereby causing cellular energy metabolism disorder and ultimately exacerbating NP cell degeneration.

### 3.2 The key glycolytic enzyme ENO1 exacerbates NP cell degeneration by inducing mitochondrial damage and metabolic imbalance

**FIGURE 2:**
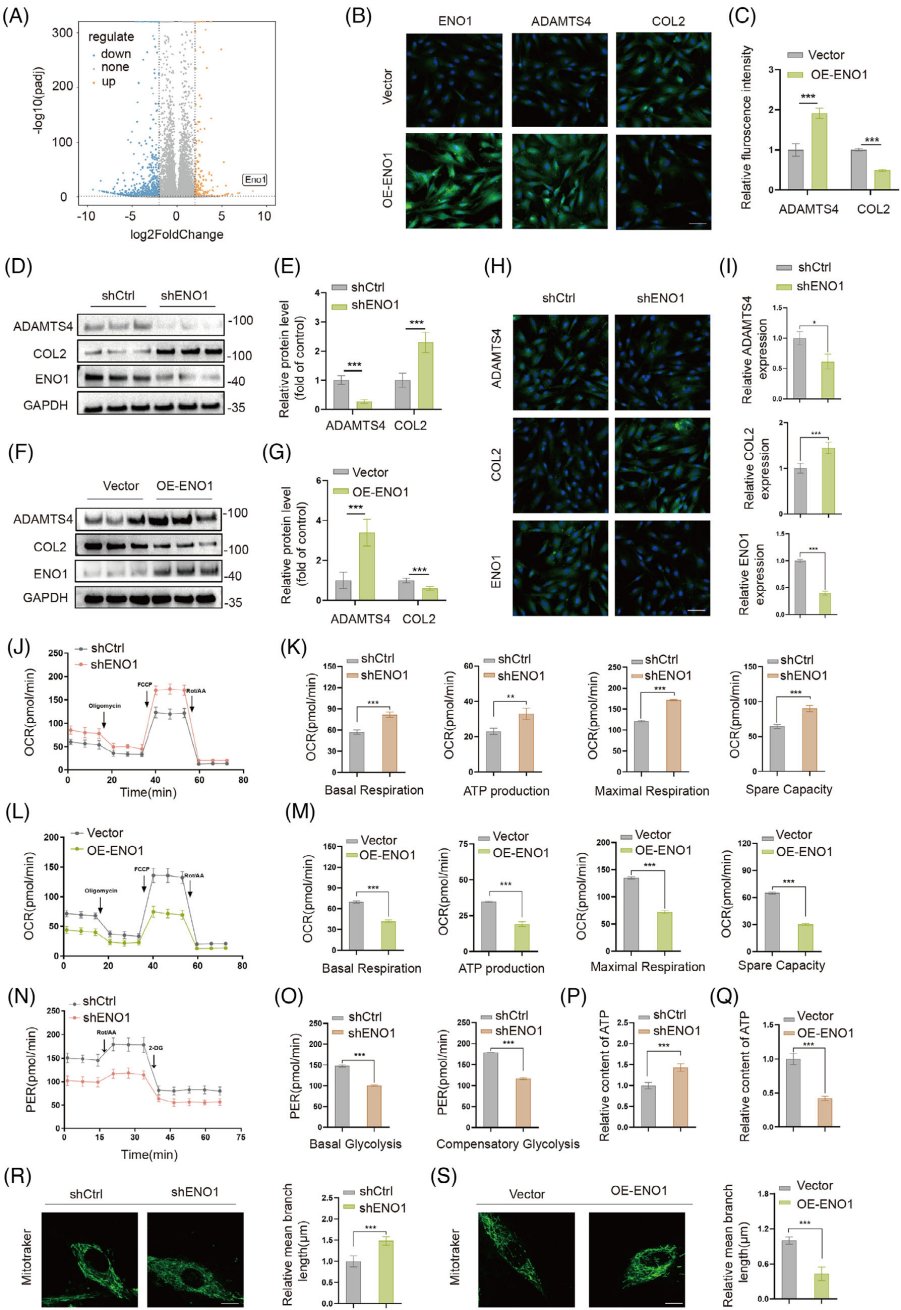
The key glycolytic enzyme ENO1 exacerbates NP cell degeneration by inducing mitochondrial damage and metabolic imbalance. (A) NP cells were divided into a control group and an IL‐1β (10 ng/mL) group, followed by differential gene volcano plot analysis. (B and C) IF analysis and quantification of ENO1, ADAMTS4, and COL2 in Vector and OE‐ENO1 NP cells (*n* = 3). Scale bars: 40 µm. (D and E) Western blot assay to detect the protein levels of TRIM25, ADAMTS4 and COL2 in shCtrl and shENO1 NP cells (*n* = 3). (F and G) Western blot analysis of TRIM25, ADAMTS4 and COL2 protein levels in Vector and OE‐ENO1 NP cells (*n* = 3). (H and I) Analysis and quantification of ENO1, ADAMTS4 and COL2 levels in shCtrl and shENO1 NP cells by IF staining (*n* = 3). Scale bars: 40 µm. (J and K) OCR measurements and mitochondrial respiration analysis in shCtrl and shENO1 NP cells (*n* = 3). (L and M) OCR profiles and mitochondrial respiration analysis in Vector and OE‐ENO1 NP cells (*n* = 3). (N and O) PER profiles and analysis of basal and compensatory glycolysis in shCtrl and shENO1 NP cells (*n* = 3). (P) Quantification of ATP content in shCtrl and shENO1 NP cells (*n* = 3). (Q) Quantification of ATP content in Vector and OE‐ENO1 NP cells (*n* = 3). (R) Representative images of Mitotracker staining to observe the mitochondrial network of shCtrl and shENO1 NP cells, and the statistical analysis of mean branch length (*n* = 3). Scale bars: 20 µm. (S) Mitotracker staining images of mitochondrial networks in Vector and OE‐ENO1 NP cells, along with quantification of mean branch length (*n* = 3). Scale bars: 20 µm. All data are expressed as the mean ± SD. One‐way ANOVA and Tukey's multiple comparisons test were used for statistical analysis. *p <  .05, **p <  .01, ***p <  .001. ADAMTS4, a disintegrin and metalloproteinase with thrombospondin motifs 4; ANOVA, analysis of variance; ATP, adenosine triphosphate; COL2, collagen type II; ENO1, enolase 1; IDD, intervertebral disc degeneration; IF, immunofluorescence; IL‐1β, interleukin‐1 beta; NP, nucleus pulposus; OCR, oxygen consumption rate; PER, proton efflux rate; SD, standard deviation.

To further investigate the mechanisms by which TRIM25 is upregulated in degenerated NP cells and regulates cellular metabolism, we next investigated its potential downstream targets. Transcriptome sequencing revealed a significant increase in the key glycolytic enzyme ENO1 following IL‐1β treatment (Figure 2A and Figure S2A), which was further validated by Wb (Figure S2B) and qPCR (Figure S2C). Knockdown of ENO1 mitigated IL‐1β‐induced degeneration, marked by increased COL2 and decreased ADAMTS4 protein expression (Figure 2D,E,H,I). Conversely, ENO1 overexpression worsened the degenerative phenotype (Figure 2B,C,F,G). Collectively, these results indicate that ENO1 promotes NP cell degeneration.

We next investigated whether ENO1 regulates mitochondrial function and energy metabolism in NP cells. Using Seahorse analysis, we found that decreased ENO1 significantly increased basal respiration, ATP production, maximal respiration, and spare respiratory capacity (Figure 2J,K). Conversely, ENO1 overexpression impaired oxidative phosphorylation (Figure 2L,M). As expected for a glycolytic enzyme, ENO1 knockdown reduced basal glycolysis and compensatory capacity (Figure 2N,O), while its overexpression enhanced glycolytic flux (Figure S2D–E). Notably, these metabolic shifts resulted in opposite changes in total intracellular ATP levels (Figure 2P,Q), suggesting that ENO1‐driven glycolysis fails to compensate for mitochondrial dysfunction.

Fluorescence imaging revealed that ENO1 knockdown promoted mitochondrial fusion, reflected by elongated tubules and increased branch length (Figure 2R). In contrast, ENO1 overexpression induced fragmentation, leading to a punctate morphology with shortened branches (Figure 2S). Collectively, these results demonstrate that ENO1, which is upregulated in degenerated NP cells, drives glycolysis while simultaneously impairing mitochondrial respiration and integrity.

### 3.3 TRIM25 regulates ENO1 degradation via K48‐linked polyubiquitination in a manner independent of its E3 ligase activity

**FIGURE 3:**
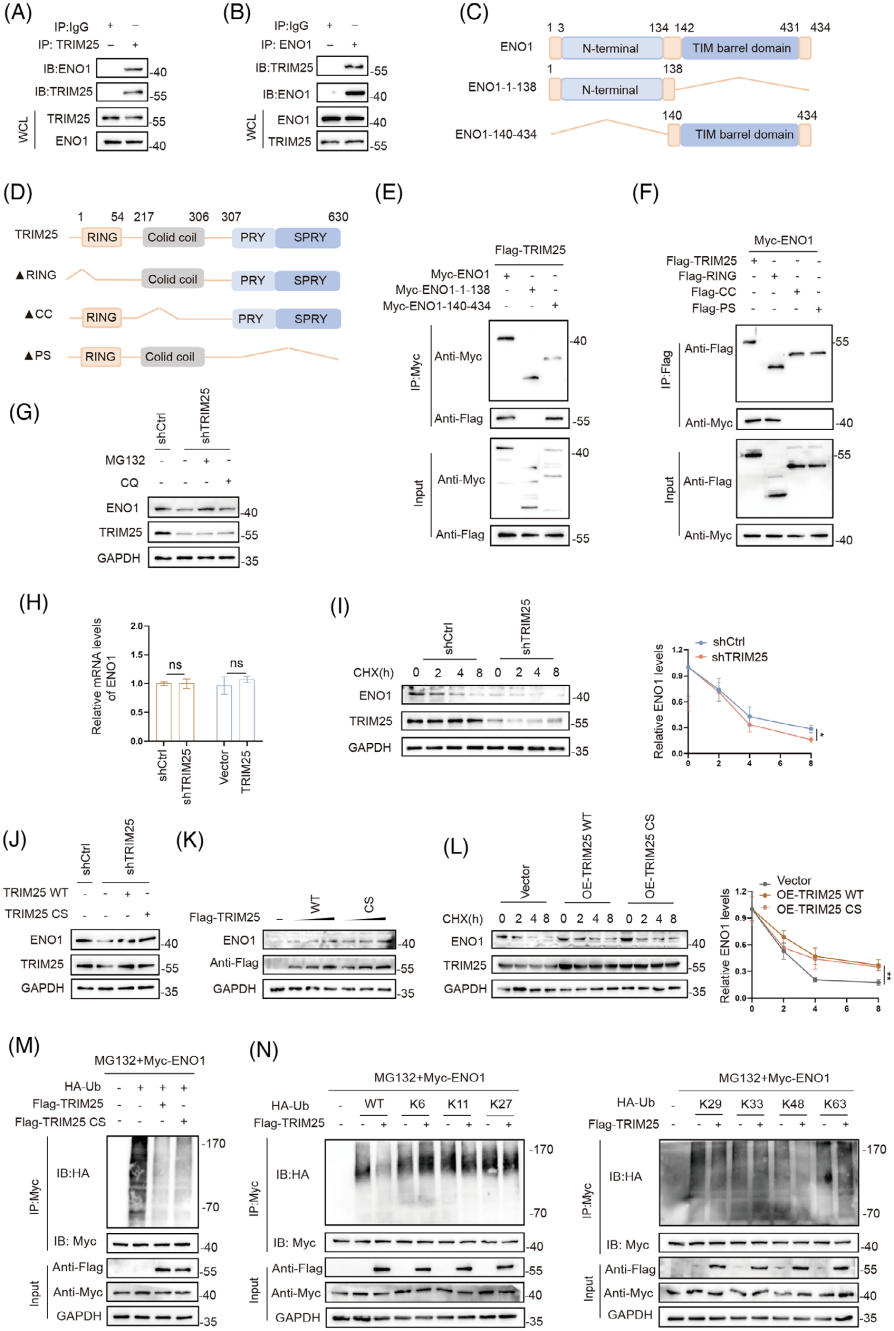
TRIM25 regulates ENO1 degradation via K48‐linked ubiquitination and an E3 independent function. (A and B) NP cell lysates were immunoprecipitated with IgG, TRIM25 (A) or ENO1 (B) antibodies, followed by immunoblotting with TRIM25 or ENO1 antibodies. (C and D) Schematic diagrams of FL and domain structure of ENO1 (C) and TRIM25 (D). (E) NP cells were co‐expressing Flag‐TRIM25 and Myc‐FL ENO1 or its domain overexpression plasmid. Cell lysates were subjected to IP, followed by western blot. F: NP cells were co‐transfected with Myc‐ENO1 and Flag‐labelled FL TRIM25 or domain overexpression plasmid. Cell lysates were subjected to IP, followed by western blot. (G) TRIM25 and ENO1 levels were analysed by western blot. (H) RT‐qPCR analyses of ENO1 mRNA levels in NP cells transfected as indicated. (I) Control and TRIM25‐knockdown NP cells were treated with CHX (50 µg/mL) and harvested at indicated time points (*n* = 3). Protein lysates were subjected to western blot. Quantification of ENO1 levels relative to GAPDH are shown. (J) NP cells transfected as indicated were analysed by western blot using anti‐TRIM25 and anti‐ENO1 antibodies. (K) NP cells transfected with Flag‐labelled wild‐type TRIM25 or TRIM25‐C50S were analysed by western blot with antibody against Flag and ENO1. (L) NP cells were co‐transfected with Myc‐ENO1 and Flag‐labelled WT TRIM25 or TRIM25‐C50S, and then treated with CHX (50 µg/mL) and collected at the indicated times (*n* = 3). Cell lysates were subjected to western blot with antibodies against Myc and Flag. (M) NP cells co‐expressing HA‐Ub, Myc‐ENO1 and Flag‐ WT TRIM25 or TRIM25‐C50S were treated with MG‐132 (20 µM, 8 h) before harvest. Lysates were subjected to d‐IP and western blot. (N) NP cells were co‐transfected with Myc‐ENO1, Flag ‐TRIM25, WT Ub or different Ub chain overexpression plasmids and treated with MG‐132 (20 µM for 8 h) before collecting. Cell lysates were subjected to d‐IP and western blot assays. All data are expressed as the mean ± SD. One‐way ANOVA and Tukey's multiple comparisons test were used for statistical analysis. *p <  .05, **p <  .01, ***p <  .001. CHX, cycloheximide; d‐IP, denatured immunoprecipitation; ENO1, enolase 1; FL, full‐length; GAPDH, glyceraldehyde‐3‐phosphate dehydrogenase; IgG, immunoglobulin G; IP, immunoprecipitation; MG132, proteasome inhibitor MG‐132; NP, nucleus pulposus; qPCR, quantitative polymerase chain reaction; TRIM25, tripartite motif containing 25; Ub, ubiquitin.

To elucidate the underlying mechanism between TRIM25 and ENO1, we performed Co‐IP experiments, as shown in Figure 3A,B, the result confirmed the specific interaction between TRIM25 and ENO1. To map the precise interaction domains, we generated truncated constructs. ENO1 was divided into its N‐terminal (1–138) and C‐terminal (140–434) domains (Figure 3C).^39^ Co‐IP assays revealed that the C‐terminal domain of ENO1 was sufficient for binding to TRIM25 (Figure 3E). Conversely, domain mapping of TRIM25, comprising RING, Coiled‐Coil (CC), and PRY‐SPRY (PS) domains (Figure 3D), demonstrated that the RING domain of TRIM25 mediates this interaction (Figure 3F). These results establish that ENO1 interacts with TRIM25 specifically through its C‐terminal domain binding to the RING domain of TRIM25.

Next, we sought to investigate the effect of TRIM25 on ENO1 expression. As shown in Figure 3G, the ENO1 protein level was decreased in NP cells with TRIM25 knockdown, which could be reversed by the proteasome inhibitor MG132 but not the lysosome inhibitor CQ, which indicates TRIM25 mediates ENO1 degradation primarily via the proteasomal pathway. Consistently, qPCR analysis revealed no significant changes in ENO1 mRNA levels upon either TRIM25 overexpression or knockdown (Figure 3H), ruling out the possibility of TRIM25 regulating ENO1 at the transcriptional level. Moreover, as shown in Figure 3I, in cells with TRIM25 knockdown, the degradation rate of ENO1 was significantly accelerated, and its half‐life was markedly shortened, directly proving that TRIM25 is crucial for maintaining ENO1 protein stability.

To investigate the role of TRIM25's E3 ligase activity in regulating ENO1, we generated a ligase‐dead mutant (TRIM25‐C50S). As shown in Figure 3J,K, both wild‐type TRIM25 (TRIM25‐WT) and TRIM25‐C50S reversed the decrease in ENO1 protein levels observed in TRIM25‐knockdown cells. Consistently, protein half‐life assays (Figure 3L) revealed that overexpression of either TRIM25‐WT or TRIM25‐C50S slowed the degradation rate of ENO1. These results indicate that TRIM25 possesses a function in stabilizing ENO1 that is independent of E3 enzymatic activity.

To explore the mechanisms involved, we performed in vitro ubiquitination assays. As shown in Figure 3M, both TRIM25‐WT and the ligase‐dead TRIM25‐C50S mutant reduced ENO1 ubiquitination levels, confirming that TRIM25 possesses a novel function independent of its E3 ligase activity that inhibits ENO1 ubiquitination. We further demonstrated that TRIM25 primarily decreases K48‐linked polyubiquitination of ENO1 (Figure 3N), which typically targets proteins for proteasomal degradation. In summary, these findings confirmed that TRIM25 inhibits ENO1 degradation via de‐K48‐linked ubiquitination, which is independent of its enzymatic activity.

### 3.4 TRIM25 promotes IDD progression and impairs energy metabolism in an ENO1‐dependent manner

**FIGURE 4:**
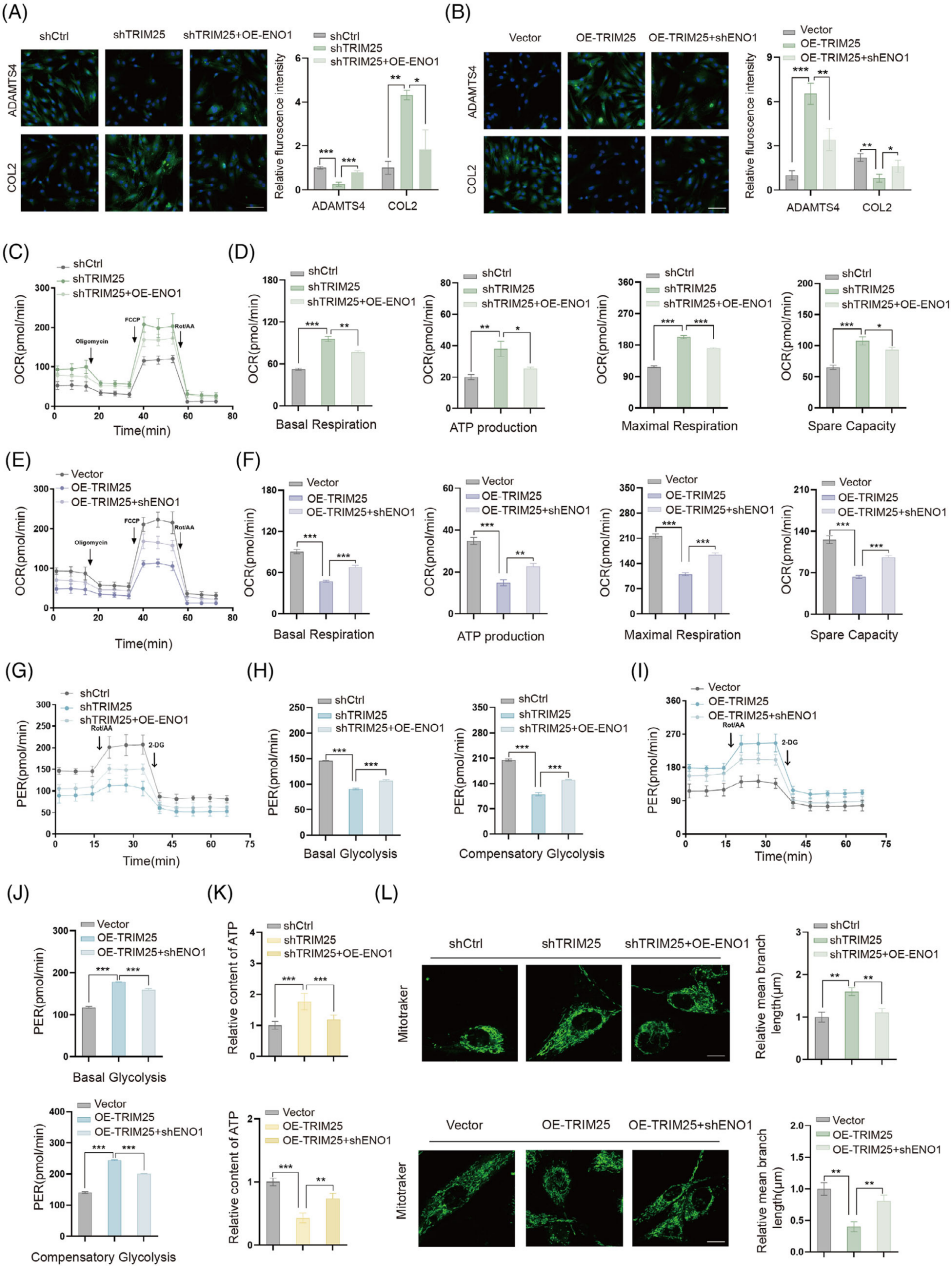
TRIM25 promotes IDD progression and impairs energy metabolism in an ENO1‐dependent manner. (A and B) IF analysis and quantification of ADAMTS4 and COL2 in NP cells transfected as indicated (*n* = 3). Scale bars: 40 µm. (C and D) OCR measurements and mitochondrial respiration analysis in shCtrl, shTRIM25, and shTRIM25+ OE‐ENO1 NP cells (*n* = 3). (E and F) OCR profiles and mitochondrial respiration analysis in Vector, OE‐TRIM25 and OE‐TRIM25+shENO1 NP cells (*n* = 3). (G and H) PER profiles and analysis of basal and compensatory glycolysis in shCtrl, shTRIM25 and shTRIM25+ OE‐ENO1 NP cells (*n* = 3). (I and J) PER profiles and analysis of basal and compensatory glycolysis in Vector, OE‐TRIM25 and OE‐TRIM25+shENO1 NP cells (*n* = 3). (K) Quantification of ATP content in NP cells transfected as indicated (*n* = 3). (L) Mitotracker staining images of mitochondrial networks in NP cells transfected as indicated, with quantification of mean branch length (*n* = 3). Scale bars: 20 µm. All data are expressed as the mean ± SD. One‐way ANOVA and Tukey's multiple comparisons test were used for statistical analysis. *p <  .05, **p <  .01, ***p <  .001. ADAMTS4, a disintegrin and metalloproteinase with thrombospondin motifs 4; ANOVA, analysis of variance; ATP, adenosine triphosphate; COL2, collagen type II; ENO1, enolase 1; IDD, intervertebral disc degeneration; IF, immunofluorescence; NP, nucleus pulposus; OCR, oxygen consumption rate; PER, proton efflux rate; SD, standard deviation; TRIM25, tripartite motif containing 25.

To further verify that TRIM25 functions through ENO1 during IDD progression, we performed rescue experiments in NP cells. As shown in Figures 4A,B and S3A, TRIM25 knockdown improved ECM metabolism (increased COL2, decreased ADAMTS4), which could be reversed by ENO1 overexpression. Conversely, the exacerbation of ECM degradation caused by TRIM25 overexpression was rescued by ENO1 knockdown. These results confirm that ENO1 acts as a critical downstream of TRIM25 in regulating ECM homeostasis.

To assess the effects of TRIM25‐ENO1 axis on energy metabolism, we performed Seahorse analysis. TRIM25 knockdown enhanced mitochondrial respiration (basal/maximal respiration, ATP production, spare capacity) (Figure 4C,D) and suppressed glycolysis (Figure 4G,H), while ENO1 overexpression reversed these effects. Conversely, TRIM25 overexpression suppressed respiration and enhanced glycolysis, which were reversed by ENO1 knockdown (Figure 4E,F,I,J). Consistently, TRIM25 knockdown promoted mitochondrial fusion (increased branch length) and elevated ATP levels, whereas TRIM25 overexpression caused mitochondrial fragmentation and ATP reduction, which were rescued by modulating ENO1 expression (Figure 4K,L). Collectively, these rescue experiments confirm that ENO1 serves as the key downstream of TRIM25, regulating the ECM metabolism, mitochondrial function, glycolysis and ATP production in NP cell degeneration.

### 3.5 TRIM25 enables USP7 to recognize and deubiquitinate ENO1

**FIGURE 5:**
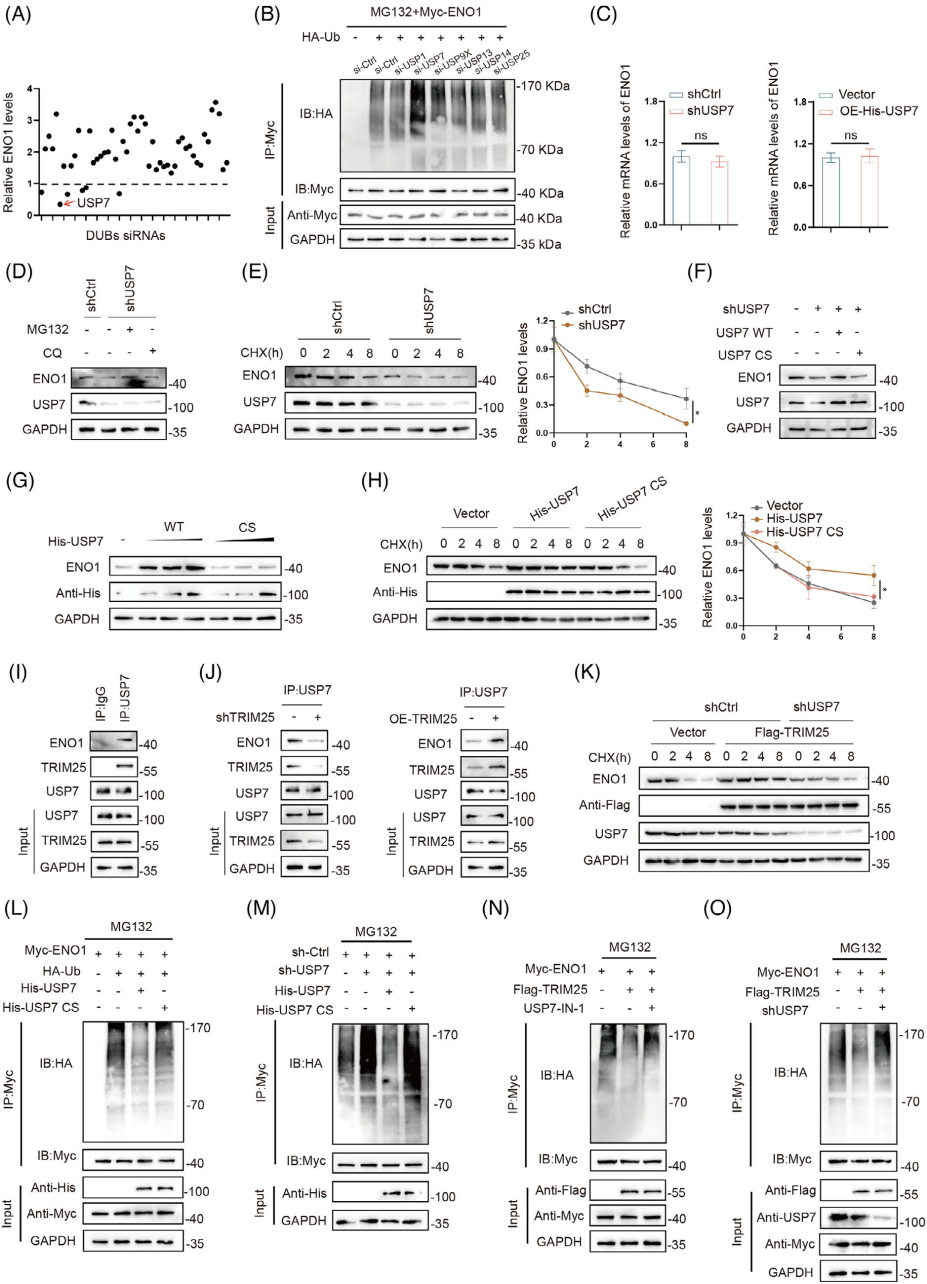
TRIM25 enables USP7 to deubiquitinate and stabilize ENO1. (A) 51 siDUBs identified six candidates, red arrow indicates USP7. (B) NP cells were co‐transfected as indicated and treated with MG‐132 before collecting. Cell lysates were subjected to d‐IP and western blot assays. (C) qPCR analyses of ENO1 mRNA levels in NP cells transfected as indicated (*n* = 3). (D) NP cells transfected as indicated were treated with or without the MG‐132 (20 µM for 8 h) or CQ (20 µM), and the expressions of USP7 and ENO1 were analysed by western blot. (E) NP cells transfected with or without USP7 shRNA, and then treated with CHX (50 µg/mL) and collected at the indicated times (*n* = 3). Cell lysates were subjected to western blot. (F) IB analysis of ENO1 levels in NP cells transduced with USP7 shRNA, together with either USP7 WT or USP7 C223S. (G) Increasing amounts of His‐ USP7 (WT or C223S) were transfected into NP cells, and cell lysates were analysed by IB. (H) NP cells were co‐transfected with His‐ USP7 WT or C223S, treated with CHX, collected at the indicated times, and then subjected to IB (*n* = 3). Quantification of ENO1 levels relative to GAPDH is shown. (I) Cell lysates of NP cells were immunoprecipitated with IgG or USP7 antibodies, and immunoblot assays were performed using USP7, TRIM25, or ENO1 antibodies. (J) NP cells were transfected as indicated and treated with MG‐132 before collecting. Cell lysates were subjected to IP, followed by western blot with the indicated antibodies. (K) NP cells were transfected as indicated and cell lysates were subjected to western blot assays. (L) NP cells were co‐transfected with HA‐Ub, Myc‐ENO1, His‐labelled WT or C223S USP7, and treated with MG‐132 before collecting. Cell lysates were subjected to d‐IP and western blot assays. (M) NP cells were co‐transfected with USP7 shRNA, His‐labelled WT or C223S USP7 and treated with MG‐132 before collecting. Cell lysates were subjected to d‐IP and western blot assays. (N) NP cells were transfected as indicated and treated with USP7‐IN‐1 for 24 h before collecting. Cell lysates were subjected to d‐IP and western blot assays. (O) NP cells were transfected as indicated and treated with MG‐132 before collecting. Cell lysates were subjected to d‐IP and western blot assays. All data are expressed as the mean ± SD. One‐way ANOVA and Tukey's multiple comparisons test were used for statistical analysis. *p <  .05, **p <  .01, ***p <  .001. ANOVA, analysis of variance; CHX, cycloheximide; CQ, chloroquine; d‐IP, denatured immunoprecipitation; ENO1, enolase 1; GAPDH, glyceraldehyde‐3‐phosphate dehydrogenase; IB, immunoblot; IgG, immunoglobulin G; IP, immunoprecipitation; MG‐132, proteasome inhibitor MG‐132; NP, nucleus pulposus; qPCR, quantitative polymerase chain reaction; SD, standard deviation; TRIM25, tripartite motif containing 25; Ub, ubiquitin; USP7, ubiquitin‐specific protease 7; USP7‐IN‐1, USP7 inhibitor IN‐1.

To identify the deubiquitinating enzyme (DUB) responsible for ENO1 stability, we systematically screened common DUBs, and identified that ENO expression levels decreased after knockdown of USP7 (Figure 5A). Moreover, we conducted in vitro ubiquitination assays, and the results showed that only siUSP7 significantly increased ubiquitination level of ENO1 (Figure 5B).

Subsequent qPCR analysis showed that the mRNA level of ENO1 remained unchanged regardless of USP7 overexpression or knockdown (Figure 5C). To determine the degradation pathway of ENO1 mediated by USP7, we used the proteasome inhibitor MG132 and the lysosome inhibitor CQ. As shown in Figure 5D, MG132 treatment completely reversed the degradation of ENO1 caused by USP7 knockdown, indicating that USP7 stabilizes ENO1 by inhibiting the proteasomal pathway. Further protein half‐life experiments directly confirmed that knocking down USP7 significantly accelerated the degradation rate of the ENO1 protein (Figure 5E). Additionally, USP7‐C223S failed to stabilize ENO1 (Figure 5F–H), proving that the deubiquitinating enzyme activity of USP7 is crucial for its function in protecting ENO1.

To elucidate the regulatory relationship among USP7, ENO1 and TRIM25, we performed Co‐IP assays. Endogenous immunoprecipitation indicated that these three proteins exist in a common complex (Figure 5I). Notably, TRIM25 knockdown weakened, whereas TRIM25 overexpression enhanced the interaction between USP7 and ENO1 (Figure 5J), indicating that TRIM25 functionally facilitates the formation of the USP7‐ENO1 complex.

Next, we conducted protein half‐life analysis, as shown in Figures 5K and S4A, the stabilizing effect of TRIM25 overexpression on ENO1 was abolished upon USP7 knockdown. Ubiquitination assays showed that overexpressing wild‐type USP7 reduced ENO1 ubiquitination, whereas USP7 knockdown or expression of USP7‐C223S increased the ENO1 ubiquitination (Figure 5L,M). Critically, the deubiquitinating effect of TRIM25 overexpression on ENO1 was completely reversed by pharmacological inhibition of USP7 (USP7‐IN‐1) or shUSP7 (Figure 5N,O). Collectively, these findings establish that the deubiquitination and stabilization of ENO1 by USP7 is critically promoted through its TRIM25‐facilitated interaction with ENO1.

### 3.6 TRIM25‐USP7‐ENO1 signalling axis drives IDD in vivo

**FIGURE 6:**
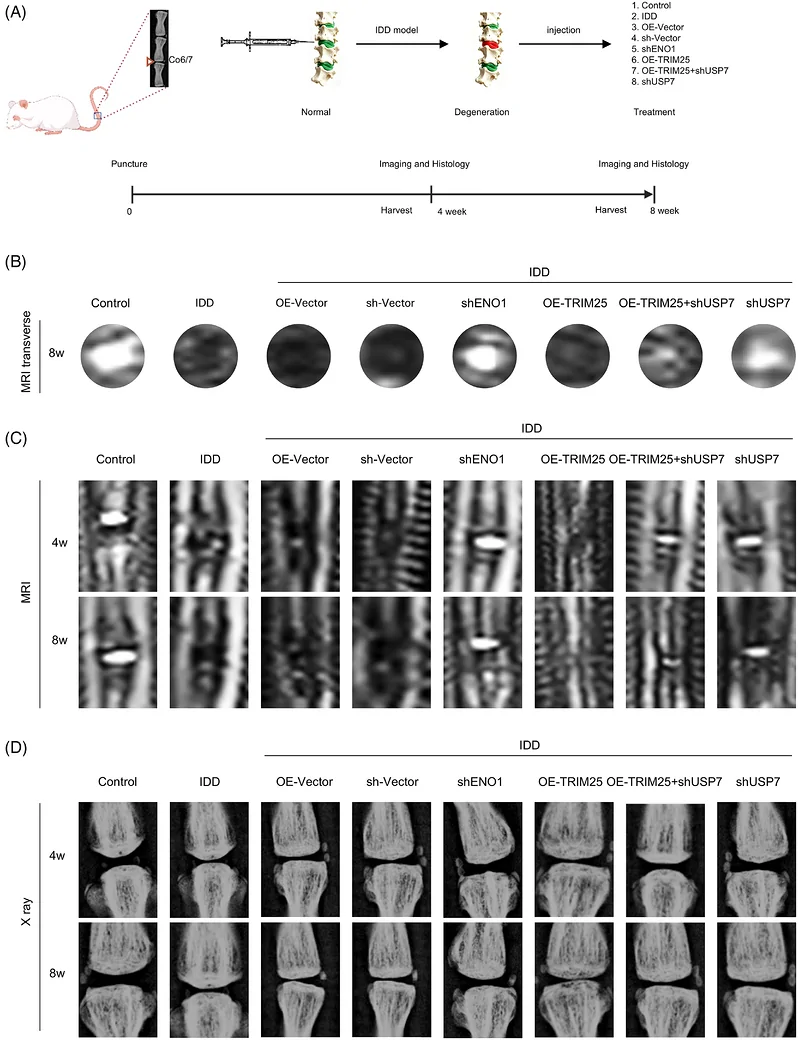
TRIM25‐USP7‐ENO1 signalling axis drives IDD in vivo. (A) Schematic diagram of the animal experimental design. (B) Representative cross‐sectional MRI scans obtained at 8 weeks post‐intervention (*n* = 6). (C) Representative MRI scans obtained at 4‐ and 8‐weeks post‐intervention (*n* = 6). (D) Representative X‐ray images of rat tails at 4‐ and 8‐weeks post‐intervention (*n* = 6). All data are expressed as the mean ± SD. One‐way ANOVA and Tukey's multiple comparisons test were used for statistical analysis. *p < 0.5, **p <  .01, ***p <  .001. ANOVA, analysis of variance; ENO1, enolase 1; IDD, intervertebral disc degeneration; MRI, magnetic resonance imaging; SD, standard deviation; TRIM25, tripartite motif containing 25; USP7, ubiquitin‐specific protease 7.

To investigate the role of the TRIM25‐USP7‐ENO1 axis during IDD progression in vivo, we established a rat tail vertebral disc needle puncture model (Figure 6A). Furthermore, the overexpression and knockdown efficiencies following viral transduction were verified by IF staining (Figure S5A). MRI T2‐weighted imaging (Figure 6B,C and Figure S5B,C) revealed a marked reduction in NP intensity in the IDD group, which was partially reversed upon knockdown of ENO1 or USP7. Notably, TRIM25 overexpression exacerbated IDD progression, which was significantly abrogated by USP7 knockdown.

Consistent with the MRI findings, X‐ray radiography analysis of the disc height index (DHI) (Figure 6D and Figure S5D,E) demonstrated a significant reduction in the IDD group, which was significantly restored in the IDD+shENO1 and IDD+shUSP7 groups. Among all experimental groups, the IDD+OE‐TRIM25 group exhibited the lowest DHI, which was significantly reversed by USP7 knockdown.

**FIGURE 7:**
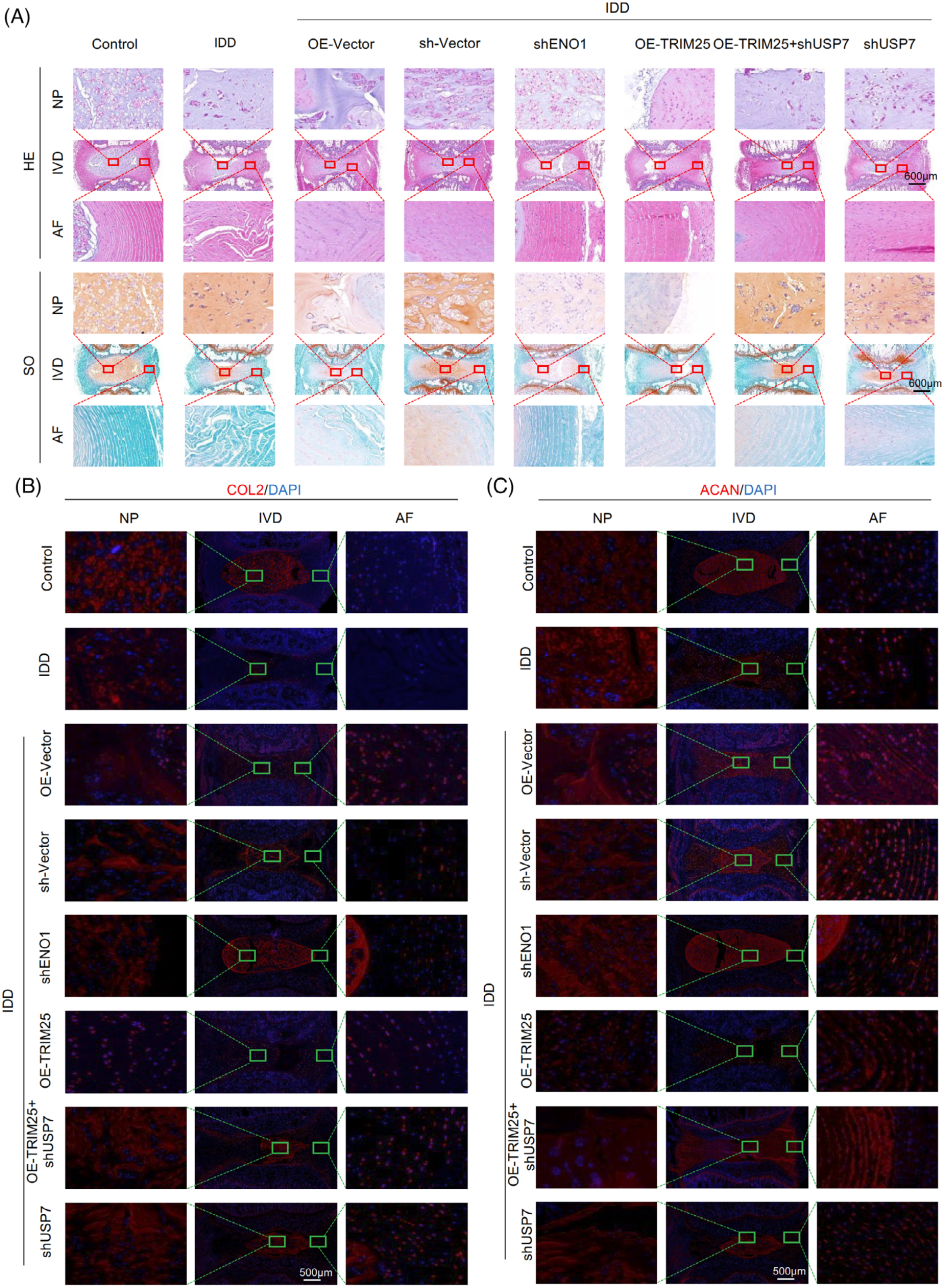
TRIM25‐USP7‐ENO1 signalling axis drives IDD in vivo. (A) Representative HE‑stained and SO‑stained images (coronal plane) of rat tail IVDs from different treatment groups at 4 weeks post‑intervention (*n* = 6). Scale bars: 600 µm. (B and C) IF detection of COL2 and ACAN at 4 weeks post‑intervention (*n* = 6). Scale bars: 500 µm. All data are expressed as the mean ± SD. One‐way ANOVA and Tukey's multiple comparisons test were used for statistical analysis. *p <  .05, **p <  .01, ***p <  .001. ACAN, aggrecan; ANOVA, analysis of variance; COL2, collagen type II; ENO1, enolase 1; HE, haematoxylin and eosin; IDD, intervertebral disc degeneration; IF, immunofluorescence; IVDs, intervertebral discs; SD, standard deviation; SO, safranin O; TRIM25, tripartite motif containing 25; USP7, ubiquitin‐specific protease 7.

**FIGURE 8:**
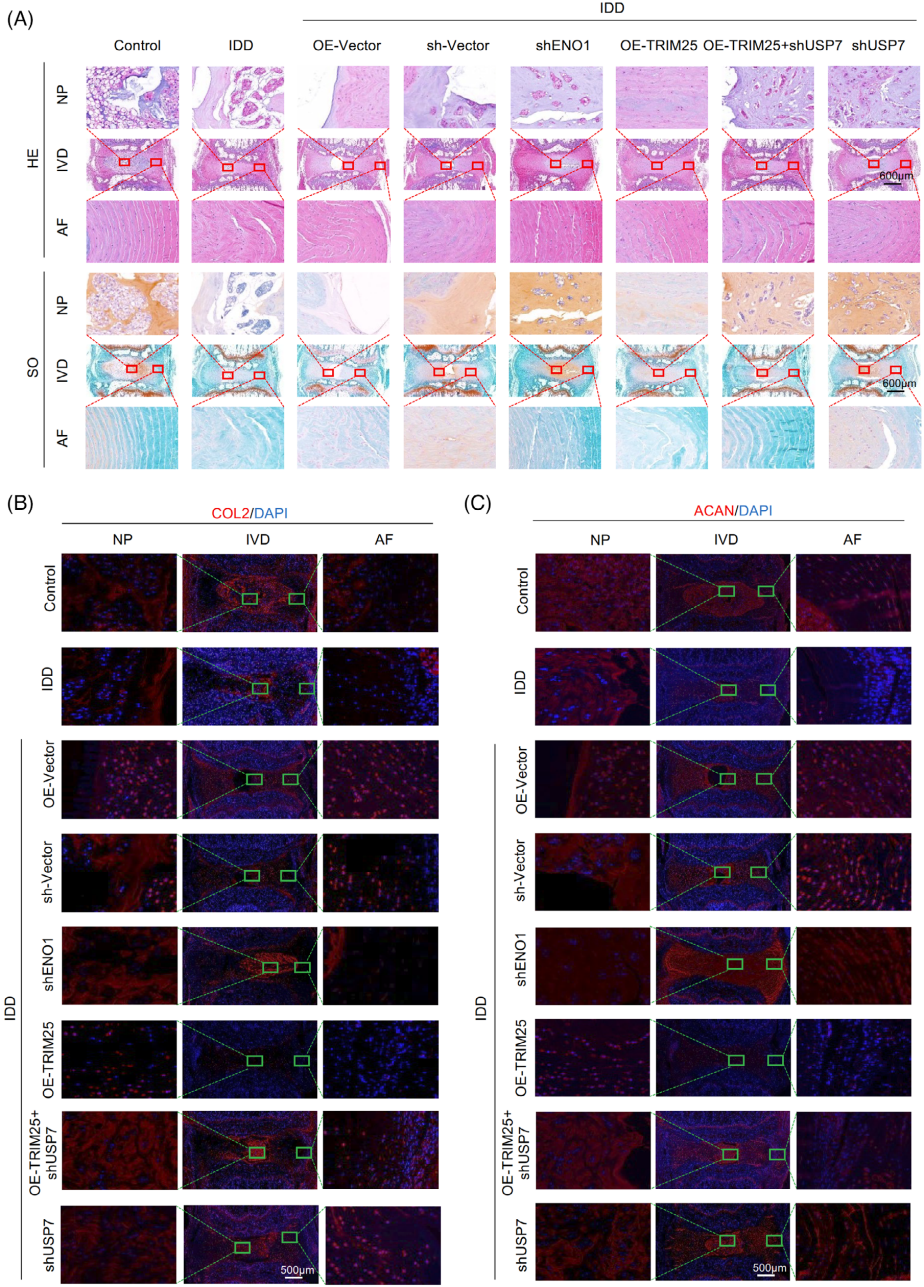
TRIM25‐USP7‐ENO1 signalling axis drives IDD in vivo. (A) Representative HE‐stained and SO‐stained images (coronal plane) of rat tail IVDs from different treatment groups at 8 weeks post‐intervention (*n* = 6). Scale bars: 600 µm. (B and C) IF detection of COL2 and ACAN at 8 weeks post‐intervention (*n* = 6). Scale bars: 500 µm. All data are expressed as the mean ± SD. One‐way ANOVA and Tukey's multiple comparisons test were used for statistical analysis. *p <  .05, **p <  .01, ***p <  .001. ACAN, aggrecan; ANOVA, analysis of variance; COL2, collagen type II; ENO1, enolase 1; HE, haematoxylin and eosin; IDD, intervertebral disc degeneration; IF, immunofluorescence; IVDs, intervertebral discs; SD, standard deviation; SO, safranin O; TRIM25, tripartite motif containing 25; USP7, ubiquitin‐specific protease 7.

H&E staining revealed (Figures 7A, 8A, S5F and S5H) that the NP tissue in the IDD group was severely damaged, which was partially alleviated upon knockdown of ENO1 or USP7. Notably, TRIM25 overexpression exacerbated tissue damage but USP7 knockdown significantly reversed this exacerbation.

SO staining for proteoglycan content showed a similar trend (Figures 7A, 8A, S5F and 5H). ECM loss was most pronounced in the IDD+OE‐TRIM25 group, whereas knockdown of ENO1 or USP7 partially preserved ECM integrity. To further evaluate the anabolism of the ECM of IVD cells at the protein level, we performed immunofluorescence staining for COL2 and ACAN on NP tissue sections. As shown in Figures 7B,C, 8B,C, S5G and S5I, compared with the sham group, the fluorescence signal intensities of COL2 and ACAN in the NP tissue of the IDD model group were significantly reduced. However, after intervention with ENO1 knockdown (IDD+shENO1) or USP7 knockdown (IDD+ shUSP7) in the IDD model, the fluorescence signals of these two key matrix components showed varying degrees of recovery. In contrast, simultaneous knockdown of USP7 with TRIM25 overexpression (IDD+OE‐TRIM25+shUSP7) partially reversed the decrease in matrix synthesis promoted by TRIM25 overexpression. These results indicate that targeting the TRIM25‐USP7‐ENO1 axis can alleviate the loss of COL2 and ACAN in the NP tissue of model rats, which is consistent with the aforementioned histological scoring results.

**FIGURE 9:**
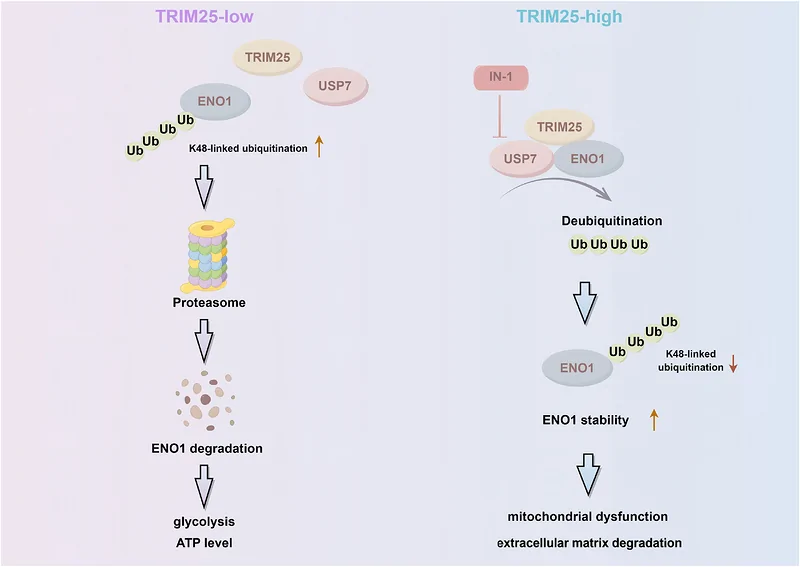
Schematic illustration of the TRIM25‐USP7‐ENO1 axis in IDD. In degenerated NP cells, TRIM25 expression is upregulated. TRIM25 acts to stabilize the glycolytic key enzyme ENO1, by facilitating its USP7‐mediated deubiquitination. Increased ENO1 stability impairs mitochondrial function, and shifts cellular energy metabolism from oxidative phosphorylation toward glycolysis. This metabolic reprogramming ultimately leads to NP cell dysfunction and ECM degradation, thereby exacerbating IDD progression. ECM, extracellular matrix; ENO1, enolase 1; IDD, intervertebral disc degeneration; NP, nucleus pulposus; TRIM25, tripartite motif containing 25; USP7, ubiquitin‐specific protease 7.

Collectively, these results establish the TRIM25‐USP7‐ENO1 axis as a critical driver of IDD pathogenesis and highlight its potential as a therapeutic target (Figure 9).

## 4 DISCUSSION

IDD is the leading cause of chronic low back pain, with complex pathological mechanisms involving multiple biological processes such as ECM imbalance, inflammatory responses, cellular senescence and energy metabolism disorders. In recent years, energy metabolism dysfunction in NP cells has been recognized as a key driver in the early occurrence and development of IDD. Our study reveals a novel molecular mechanism by which the E3 ubiquitin ligase TRIM25 and the deubiquitinase USP7 jointly regulate the protein stability of the key glycolytic enzyme ENO1, thereby playing a central role in the IDD process. Our research not only uncovered a TRIM25‐ENO1 axis linking metabolism but, more importantly, defined the TRIM25‐USP7‐ENO1 regulatory axis, in which TRIM25 enables ENO1 stabilization through USP7 (Figure 9).

TRIM25 is well‐known for the role of a ‘sentinel’ in the antiviral innate immune response. As an E3 ubiquitin ligase, TRIM25 can recognize and be activated by viral RNA‐bound RIG‐I, subsequently catalysing K63‐linked polyubiquitination modification,^16^, ^40^, ^41^ which established the core position of TRIM25 in immunology. Our study expands the research scope of TRIM25 from relatively mature immunological field to the skeletal system, IDD. We observed significant upregulation of TRIM25 at both transcript and protein levels in rat degenerated NP tissue and in vitro degeneration models. In fact, TRIM25 has been reported to be significantly induced by inflammatory cytokines or cellular stress signals in various chronic inflammatory diseases and the tumour microenvironment.^42^, ^43^ However, rather than merely describing TRIM25 upregulation in IDD, our study reveals a novel downstream target and function of TRIM25 in this process—specifically, its regulation of K48‐linked polyubiquitination of ENO1. However, unlike the conventional E3 ligases directly catalyse K48 ubiquitination of substrates to promote degradation, our data show that overexpression of TRIM25 actually led to a reduction in K48‐linked polyubiquitin chains on the ENO1 protein.

Building on this, our study further identifies a novel non‐canonical function of TRIM25 that is critical for the deubiquitination of ENO1, namely, promoting the functional interaction between ENO1 and its deubiquitinase, USP7. We confirmed the interaction between USP7 and ENO1, and found that USP7 catalyses the deubiquitination of ENO1—a process that is highly dependent on the presence of TRIM25. A precedent has also demonstrated that the E3 ligase KEAP1 can both target NRF2 for ubiquitination and degradation, and act to bring other proteins together.^44^, ^45^, ^46^, ^47^ The TRIM25‐USP7‐ENO1 regulatory axis identified in this study adds a new dimension to understanding the function of TRIM25 in IDD. Unlike previous reports that focused on the role of TRIM25 in stress response or senescence‐related pathways,^42^, ^48^ our work is the first to reveal that TRIM25 facilitates the USP7‐dependent deubiquitination and stabilization of the metabolic enzyme ENO1, thereby directly regulating glycolysis flux and ECM metabolism in NP cells. This suggests that TRIM25 may play multiple regulatory roles in the complex pathological network of IDD by acting on different substrates. Future studies need to further integrate different signalling layers to map the comprehensive regulatory network of TRIM25 in IDD.

ENO1 is the key enzyme in the glycolytic pathway that catalyses the conversion of 2‐PG to PEP. Moreover, ENO1 is also a typical ‘moonlighting protein’, performing various functions at different locations such as the cell surface and nucleus, for example, as pro‐tumorigenic effects, a plasminogen receptor, etc.^49^, ^50^, ^51^ In the field of cancer research, some studies suggest that other E3 ligases, such as F‐Box, might be involved in ENO1 degradation and affect cancer cell proliferation,^52^, ^53^, ^54^ but these studies are completely unrelated to IDD pathology. Here, we report that ENO1 is upregulated in degenerated NP cells and drives glycolysis while simultaneously impairing mitochondrial respiration and integrity, thereby exacerbating IDD progression. Furthermore, we reveal the core regulatory role of TRIM25 in controlling ENO1 protein stability and demonstrate that this regulation occurs via the K48‐linked ubiquitination pathway during IDD progression.

USP7, also known as HAUSP, is one of the most characterized members of the deubiquitinase family.^55^, ^56^ USP7 mainly regulates the cell cycle, DNA repair and apoptosis.^57^, ^58^, ^59^ Furthermore, USP7 has been reported to be involved in epigenetic regulation, immune regulation and viral infection.^55^, ^60^, ^61^ Our study confirmed that USP7, a typical deubiquitinase, plays a critical role in energy metabolism and ECM homeostasis during IDD progression. Specifically, USP7 directly recognizes, binds to, and deubiquitinates ENO1 via K48‐linked chain removal to stabilize it—a process that requires the promoting function of TRIM25.

Of course, our study has several limitations. First, the precise domain(s) or amino acid residue(s) on TRIM25 responsible for binding USP7 and ENO1 remain to be identified. This will require constructing a series of truncation and point mutants, combined with structural biology approaches, to ultimately elucidate the molecular basis of this promoting mechanism. Second, at the level of signalling regulation, it remains unclear how this functional association is precisely regulated by specific post‐translational modifications (e.g., phosphorylation)—a question that is crucial for understanding the balance ‘switch’. Finally, from a translational medicine perspective, future studies should validate whether interfering with this axis holds therapeutic potential in animal models that more closely resemble human pathology, such as rat tail disc degeneration models. Moreover, whether our findings prompt re‐evaluation of the potential application of relevant inhibitors in metabolic diseases represents an intriguing translational direction. In this context, recent work has shown that biologically rational targeted interventions can still produce unexpected in vivo toxicities, for example, a phase 1 and murine study of CD5‐KO anti‐CD5 CAR‐T cells reported prolonged immune perturbation, infection risk, autoimmune events, and tissue‐specific on‐target/off‐tumour effects.^62^ Although this study reveals the therapeutic potential of targeting the TRIM25‐USP7‐ENO1 axis in alleviating IDD, its clinical translation still faces a series of specific challenges. The primary challenge lies in developing highly selective inhibitors capable of precisely modulating this axis. Given the broad roles of TRIM25 and USP7 in various cellular processes, future drug design should focus on disrupting their specific interaction interfaces with ENO1, rather than inhibiting their catalytic activity globally, to minimize off‐target effects. Secondly, the IVD, as an avascular, hyperosmotic, and enclosed structure, poses a significant obstacle to systemic drug delivery. Developing delivery systems (such as nanocarriers or engineered exosomes) that can efficiently penetrate the AF and target NP cells is a critical direction for future research. In addition, future studies should preferably include multiple spinal segments to minimize inter‐individual variability and, where possible, perform intra‐individual comparisons across different segments. Finally, when advancing preclinical evaluation, it is essential to systematically investigate the potential long‐term effects of intervening in this axis on systemic metabolic homeostasis, immune function, and adjacent disc tissues. Overcoming these challenges will require interdisciplinary collaboration, but success may pave a new avenue for disease‐modifying therapy in IDD.

## 5 CONCLUSION

This study reveals that TRIM25 facilitates the functional engagement of the deubiquitinase USP7 with the glycolytic enzyme ENO1, leading to ENO1 stabilization. The stabilization of ENO1 by TRIM25, achieved through promoting its USP7‐mediated deubiquitination, provides novel insights into the pathogenesis of intervertebral disc degeneration.

## AUTHOR CONTRIBUTIONS

Conceptualization: Xiaoming Liu, Wenyu Zhang, Hang Feng and Linying Lai. Methodology, fabrication and investigation: Xiaoming Liu, Wenyu Zhang, Hang Feng, Linying Lai, Chen Cao, Guang Yang, Yanzheng Gao and Zhenghong Yu. Writing–original draft: Xiaoming Liu, Hang Feng, Wenyu Zhang and Linying Lai. Writing–review & editing: Bijun Wang, Hui Wang, and Bin Yu. Xiaoming Liu, Wenyu Zhang, Hang Feng and Linying Lai are co‐first authors. Bijun Wang, Hui Wang and Bin Yu are co‐corresponding authors.

## CONFLICTS OF INTEREST STATEMENT

The authors declare no conflicts of interest.

## ETHICS STATEMENT

The study was reviewed and approved by Ethics Committee of Shanghai East Hospital.

## Supporting information



Supporting Information

## Data Availability

All data generated or analysed during this study are included in this published article and its Supporting Information files.
