# Supplementary material for: TRIM25 facilitates mitochondrial dysfunction and extracellular matrix degradation by enhancing USP7‐driven ENO1 deubiquitination during intervertebral disc degeneration
Source: Clin Transl Med. 2026 Aug 31;16(9):e70785. doi: 10.1002/ctm2.70785 (PMC13528619; doi:10.1002/ctm2.70785)
Supplement: Supplementary file 1 [file CTM2-16-e70785-s001.pdf]

Table S1. Antibodies used in this study

Antibody	Manufacturer	Catalog No.
COL2	Proteintech	28459-1-AP
ADAMTS4	Proteintech	11865-1-AP
ubiquitin	Proteintech	80992-1-RR
GAPDH	Proteintech	60004-1-Ig
Flag	Cell Signaling Technology	14793
Myc	Cell Signaling Technology	2276
HA	Cell Signaling Technology	3724
His	Cell Signaling Technology	12689
TRIM25	Proteintech	12573-1-AP
ENO1	Proteintech	11204-1-AP
USP7	Proteintech	66514-1-Ig

COL2, Collagen type II; ADAMTS4, A disintegrin and metalloproteinase with thrombospondin motifs 4; ENO1, Alpha-enolase; TRIM25, tripartite motif-containing 25; USP7, ubiquitin specific peptidase 7.

Table S2. The PCR primers used in this study.

	primer sequences (forward)	primer sequences (reverse)
ENO1	5′-CACCATGGCTTTGACTACAAAACC-3′	5′-TCATGGTGATCGGAAAGCTTCACC-3′
ADAMTS4	5′-CGCTGAGTAGATTCGTGGAGAC-3′	5′-AGTTGACAGGGTTTCGGATGC-3′
COL2	5′-TCCTAAGGGTGCCAATGGTGA-3′	5′-AGGACCAACTTTGCCTTGAGGAC-3′
TRIM25	5′-AGCAGCTACAACAAGAATACACG-3′	5′-GGCTCTGTTCAATCTCCTCCT-3′

ADAMTS4, A disintegrin and metalloproteinase with thrombospondin motifs 4; COL2, Collagen type II; TRIM25, tripartite motif-containing 25; ENO1, Alpha-enolase.

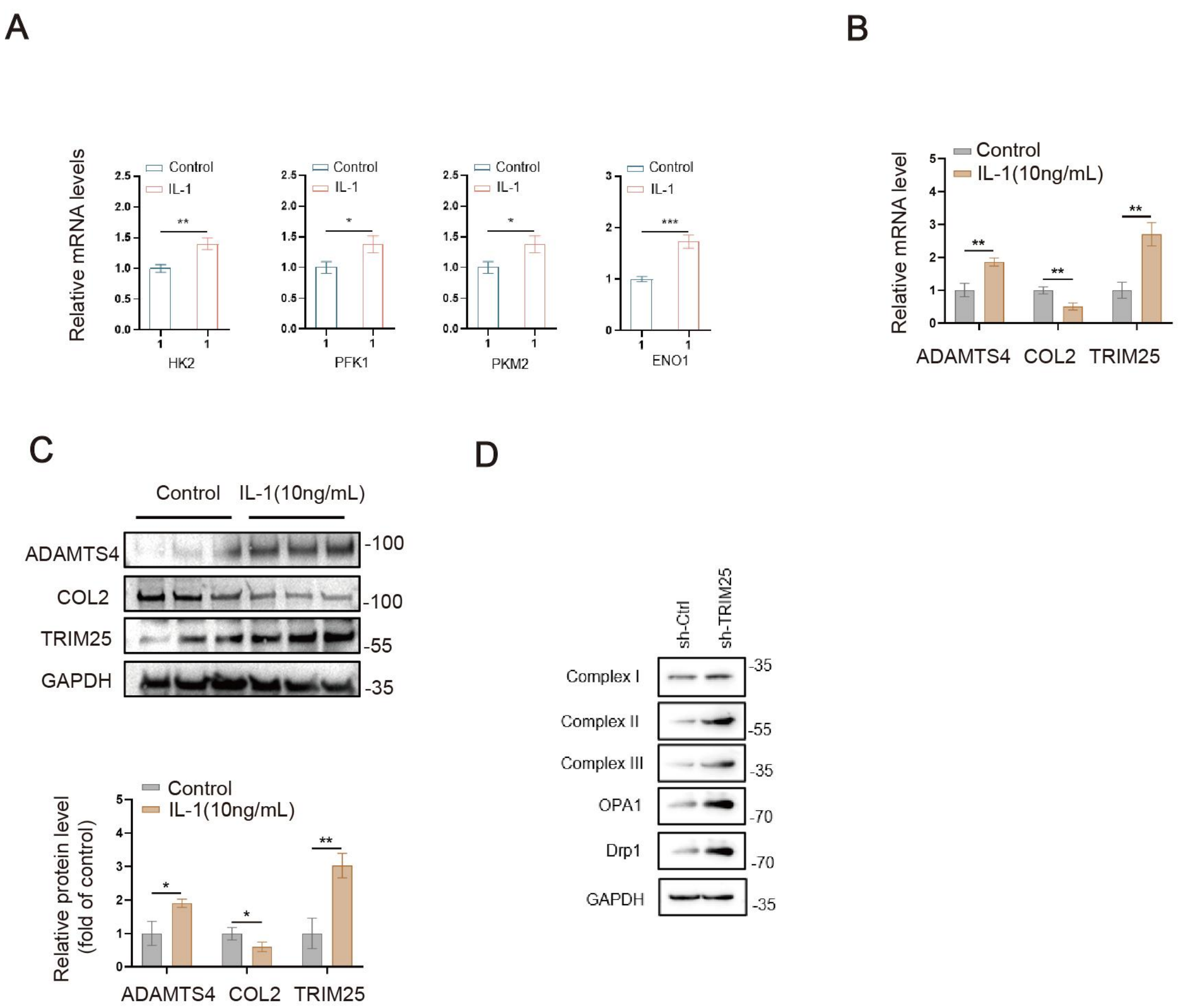

Figure S1. Mitochondrial dysfunction and IDD progression in IDD-NPs is exacerbated by TRIM25. A: qPCR analysis of glycolysis-related gene (HK2, PFK1, PKM2, ENO1) levels in NP cells treated with IL-1β for 72 hours (n = 3). B-C: West-ern blot and qPCR analysis of TRIM25, ADAMTS4 and COL2 levels in NP cells treated with IL-1β for 72 hours. D:Western blot analysis was used to detect the levels of oxidative phosphorylation and mitochondrial proteins after TRIM25 knockdown. All data are expressed as the mean ± SD. One-way ANOVA and Tukey's multiple comparisons test were used for statistical analysis. *p < 0.05, **p < 0.01, ***p < 0.001. IDD, intervertebral disc degeneration; NPs, nucleus pulposus cells; TRIM25, tripartite motif-containing protein 25; ADAMTS4, a disintegrin and metalloproteinase with thrombospondin motifs 4; COL2, collagen type II; IL-1β, interleukin-1β; qPCR, quantitative polymerase chain reaction; PFK1, phosphofructokinase 1; PKM2, pyruvate kinase M2; HK2, hexokinase 2; ENO1, enolase 1.

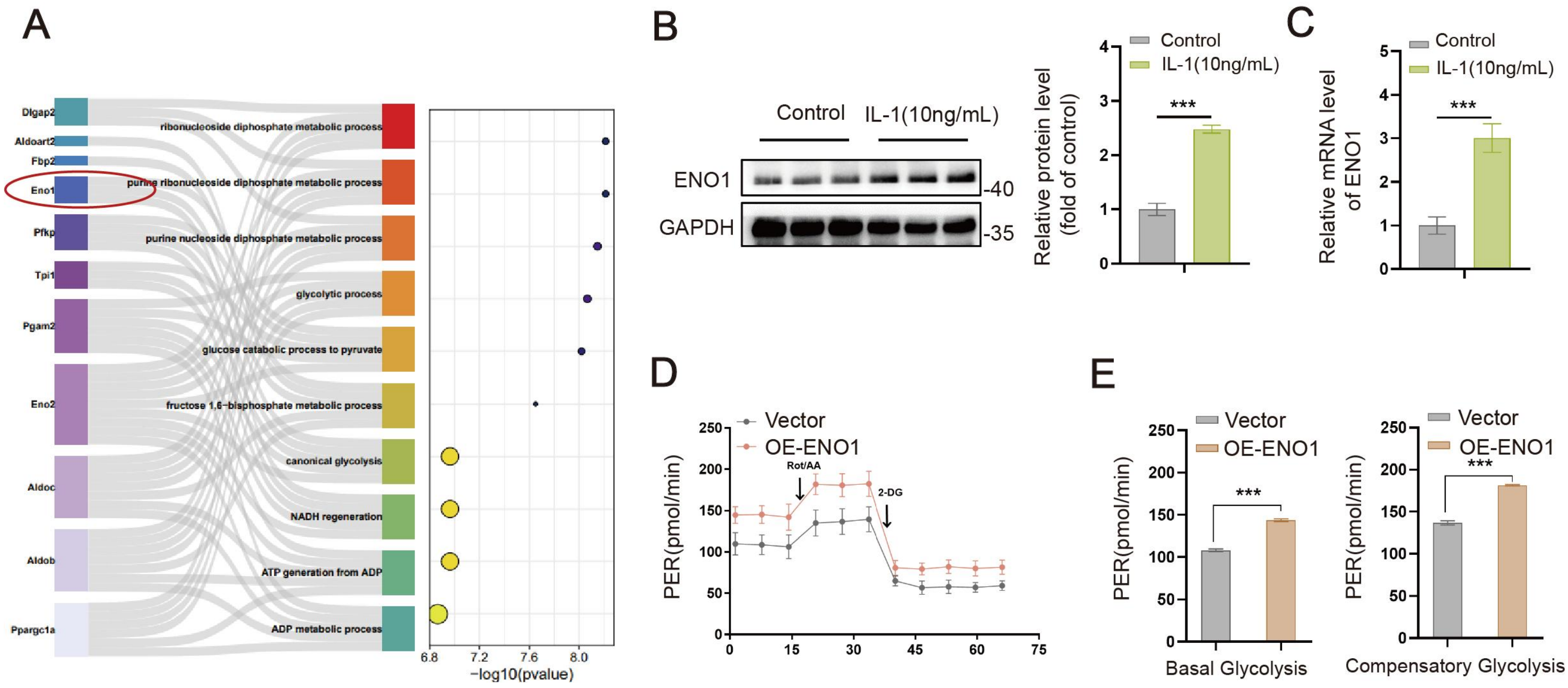

Figure S2. ENO1 promotes IDD progression and impairs mitochondrial function in NP cells. A: Nucleus pulposus cells were divided into a control group and an IDD group, followed by transcriptomic sequencing and GO enrichment analysis visualized by a mulberry plot. B-C: Western blot and qPCR analysis of ENO1 expression in NP cells treated with IL-1β for 72 hours. D-E: PER profiles and analysis of basal and compensatory glycolysis in Vector and OE-ENO1 NP cells. All data are ex-pressed as the mean ± SD. One-way ANOVA and Tukey's multiple comparisons test were used for statistical analysis. *p < 0.05, **p < 0.01, ***p < 0.001. ENO1, al-pha-enolase; IDD, intervertebral disc degeneration; NP, nucleus pulposus; GO, Gene Ontology; IL-1β, interleukin-1β; qPCR, quantitative polymerase chain reaction; PER, proton efflux rate.

A

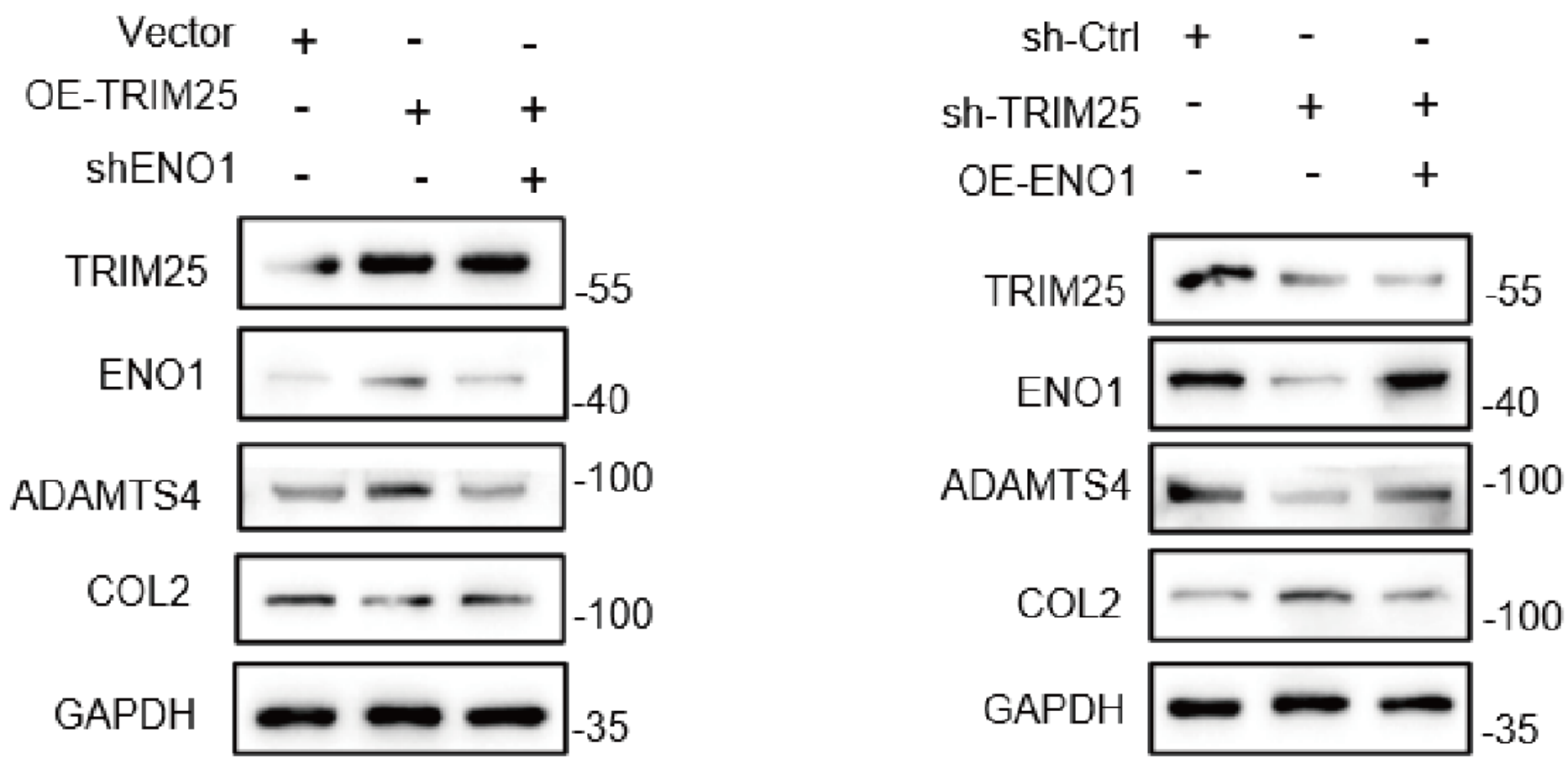

Figure S3. TRIM25 promotes IDD progression in an ENO1-dependent manner. A: Western blot analysis and quantification of ADAMTS4 and COL2 in NP cells transfected as indicated (n = 3). ADAMTS4, a disintegrin and metalloproteinase with thrombospondin motifs 4; ANOVA, analysis of variance; COL2, collagen type II; ENO1, enolase 1; IDD, intervertebral disc degeneration; NP, nucleus pulposus; SD, standard deviation; TRIM25, tripartite motif containing 25.

A

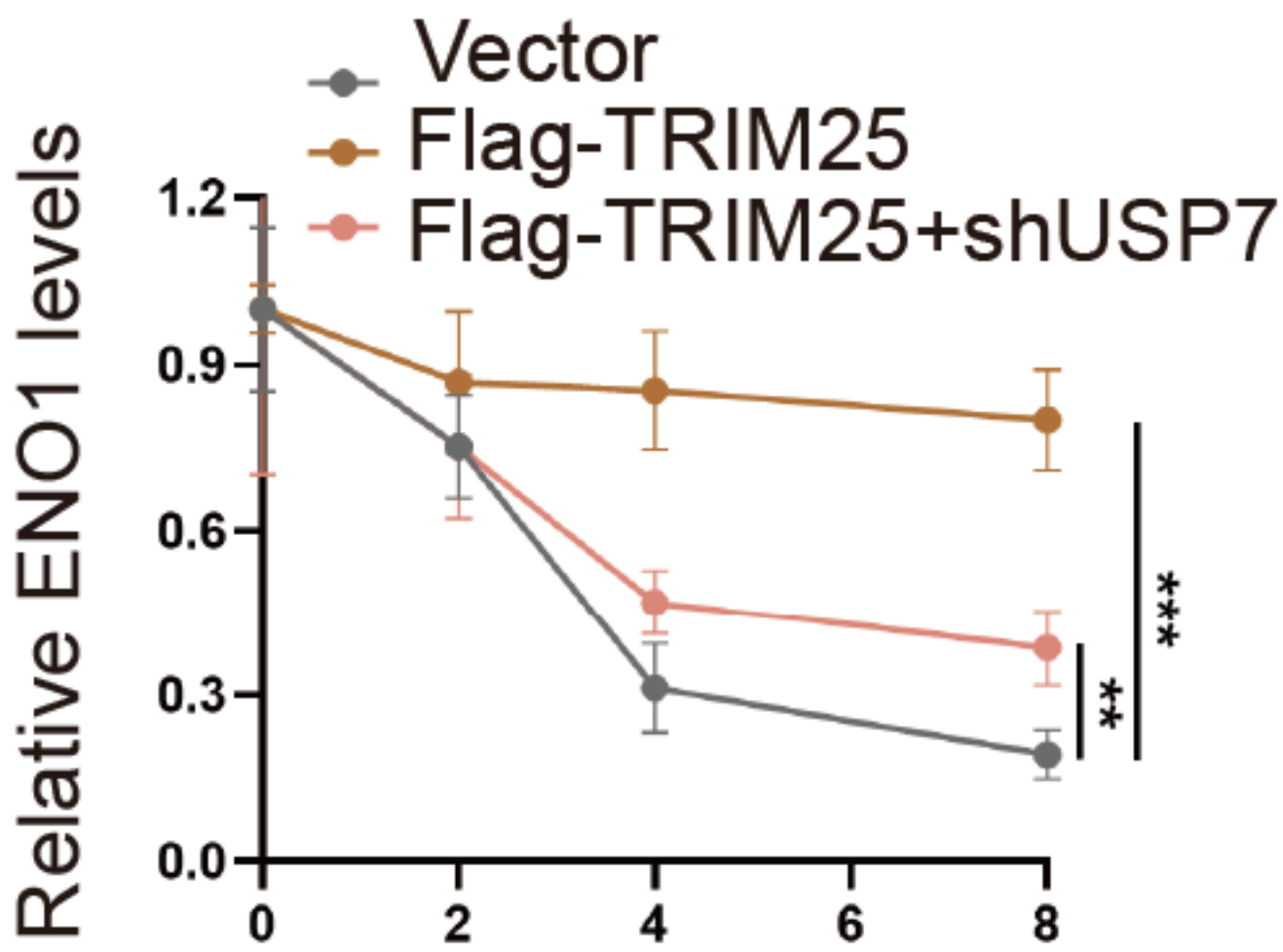

Figure S4. TRIM25 serves as a bridge to strengthen the interaction between USP7 and ENO1, modulating USP7-mediated ENO1 deubiquitination. A: Quantification of ENO1 levels relative to GAPDH is shown (n = 3). All data are expressed as the mean ± SD. One-way ANOVA and Tukey's multiple comparisons test were used for statisti-cal analysis. *$p < 0.05$, **$p < 0.01$, ***$p < 0.001$. TRIM25, tripartite motif-containing protein 25; USP7, ubiquitin-specific peptidase 7; ENO1, alpha-enolase; GAPDH, glyceraldehyde 3-phosphate dehydrogenase.

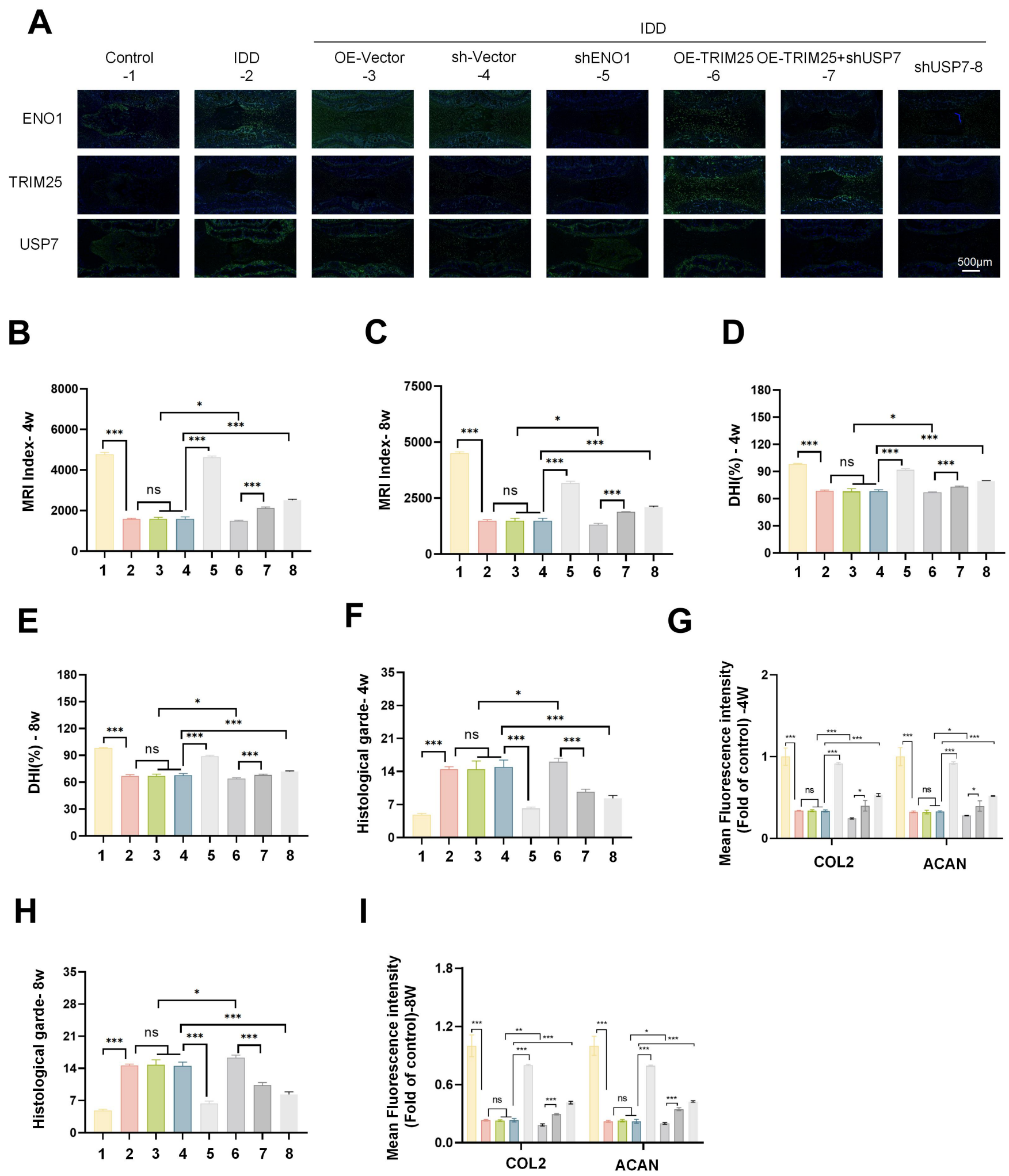

Figure S5. Observation of imaging and histological degeneration in rat tail IVDs after intervention. A: IF staining of tissues between groups for ENO1, TRIM25 and USP7. B-C: Quantitative assessment of water-content signals from representative MRI images of rat tails at 4 and 8 weeks post-intervention. D-E: Quantification of the DHI from repre-sentative X-ray images of rat tails at 4 and 8 weeks post-intervention. F-G: Histopathological scoring of rat tail IVDs corresponding to different treatment groups at 4 and 8 weeks post-intervention. H: Quantitative analysis of IF staining for COL2 and ACAN at 4 weeks post-intervention. I: quantitative analysis of COL2 and ACAN at 8 weeks post-inter-vention. All data are expressed as the mean ± SD. One-way ANOVA and Tukey's multiple comparisons test were used for statistical analysis. *p < 0.05, **p < 0.01, ***p < 0.001. IVD, intervertebral disc; COL2, collagen II; ACAN, aggrecan; IF, immunofluorescence; ECM, extracellular matrix; ENO1, Alpha-enolase; TRIM25, Tripartite motif-con-taining protein 25; USP7, Ubiquitin-specific peptidase 7; DHI, disc height index; IVD, intervertebral disc.
